# Designing a multi-epitope peptide based vaccine against SARS-CoV-2

**DOI:** 10.1038/s41598-020-73371-y

**Published:** 2020-10-01

**Authors:** Abhishek Singh, Mukesh Thakur, Lalit Kumar Sharma, Kailash Chandra

**Affiliations:** 1 Zoological Survey of India, New Alipore, Kolkata, West Bengal 700053 India; 2 Gujarat Forensic Sciences University, Gandhinagar, Gujarat 382007 India

**Keywords:** Vaccines, Computational models, Predictive medicine, Protein design, Protein function predictions, Protein structure predictions, Virtual drug screening

## Abstract

COVID-19 pandemic has resulted in 16,114,449 cases with 646,641 deaths from the 217 countries, or territories as on July 27th 2020. Due to multifaceted issues and challenges in the implementation of the safety and preventive measures, inconsistent coordination between societies-governments and most importantly lack of specific vaccine to SARS-CoV-2, the spread of the virus that initially emerged at Wuhan is still uprising after taking a heavy toll on human life. In the present study, we mapped immunogenic epitopes present on the four structural proteins of SARS-CoV-2 and we designed a multi-epitope peptide based vaccine that, demonstrated a high immunogenic response with a vast application on world’s human population. On codon optimization and in-silico cloning, we found that candidate vaccine showed high expression in *E. coli* and immune simulation resulted in inducing a high level of both B-cell and T-cell mediated immunity. The results predicted that exposure of vaccine by administrating three injections significantly subsidized the antigen growth in the system. The proposed candidate vaccine found promising by yielding desired results and hence, should be validated by practical experimentations for its functioning and efficacy to neutralize SARS-CoV-2.

## Introduction

Coronavirus disease 2019 (COVID-19) is one of the most potent pandemics characterized by respiratory infections in Human^1^. As per the latest report of World Health Organization (WHO) dated July 27th 2020, the worldwide coverage of COVID-19 exceeded 16,114,449 cases with mortality of 646,641 deaths individuals from 217 countries, areas or territories^2^. The outbreak of COVID-19 initially emerged at Wuhan city of Hubei Province of People Republic of China in the early December 2019^3^, and the report of severe pneumonic patients with unknown etiology of symptoms was released by the Chinese Center for Disease Control on December 31st 2019^4^. Later on, the novel severe acute respiratory syndrome coronavirus 2 (SARS-CoV-2) was confirmed as the causative agent for the clusters of pneumonic cases by the health professionals. Further investigations on the origin of infection possibly linked the SARS-CoV-2 to a seafood market in the Wuhan city, China which got spread to a large extent of human population in a short time span^5^. By the time, scientists/ health professionals paid a sincere note to understand the mode of transmission of SARS-CoV-2, it already infected a large number of people in various countries, by exhibiting a high transmission rate in comparison to the earlier human CoV^6^. This led the WHO to declare COVID-19 as a Public Health Emergency of International Concern (PHEIC) on January 30th 2020^7^.

Until the outbreak of SARS in 2002 and 2003, coronaviruses are considered mild pathogenic virus causing respiratory and intestinal infections in animals and humans^8–12^. With a decade long pandemic of SARS, another potent pathogenic coronavirus, i.e., Middle East Respiratory Syndrome Coronavirus (MERS-CoV) appeared explicitly in the Middle Eastern countries^13^. Extensive studies on these two coronaviruses have led to an understanding that they are genetically diverse but likely to be originated from bats^14,15^. Apart from these two viruses, four other coronaviruses, i.e. HKU1, 229E, OC43 and NL63 are commonly detected in Humans^16,17^. Among all the coronaviruses reported so far, SARS-CoV, MERS-CoV, and recently emerged SARS-CoV-2 have relatively high pathogenicity and fast mutagenic abilities^18^. SARS CoV alone was responsible for 8422 positive cases and 919 probable fatalities over a vast population of 32 countries^19^. With the first mortality case reported in 2012, 2496 cases were found positive for MERS-CoV, and 868 deaths were reported from 27 countries^19^. Both of these pandemics were considered as highly pathogenic in the last two decades until the outbreak of SARS-CoV-2 in December 2019. SARS-CoV-2 has already spread in 217 countries and caused 646,641 deaths since its outbreak in December 2019^2^. As in the current scenario, several countries, e.g. USA, Brazil, India, Russia, South Africa, Mexico, Peru, Chile, Spain, United Kingdom etc. have already experienced or experiencing the most fatal problem of community spread of SARS-CoV-2. Though the estimated mortality rate due to SARS-CoV-2 is about 3.6% in China and about 1.5% outside China^20^, the problem is grave and need to be respond urgently to curb the rapid loss of human life.

Coronaviruses are broadly divided into four genera, Alpha-CoV, Beta-CoV, Gamma-CoV and Delta-CoV, where Alpha-CoV and Beta-CoV are only reported to infect mammals but Gamma-CoV and Delta-CoV are also capable of infecting both mammals and birds^21^. An extensive study of their biology suggests that they are non-segmented enveloped viruses with a positive-sense single-stranded RNA of 26–32 kb size^16^. Some of the recent research indicated that SARS-CoV-2 has an identical genomic organization as of Beta-CoV, and thus the genomic structure of SARS-CoV-2 follows 5′-leader-UTR-replicase- spike-envelope-membrane-nucleocapsid and 3′ UTRpoly (A) tail with the subsequent species-specific accessory gene at 3′ terminal of the viral genome^22^. The structural glycoprotein includes Spike/Surface (S), Envelop (E), Membrane (M) and Nucleocapsid (N). The S glycoprotein is reported to have a crucial role in the virus transmission as the receptor binding capability and entry to the host cell is regulated by the expression of S glycoprotein^23^. The E and M glycoproteins are responsible for viral assembly and N glycoprotein is necessary for RNA genome synthesis^23^. The complex genetic makeup and high mutation rate of SARS-CoV-2 requires the strategic development of a vaccine by targeting all the structural proteins. Unfortunately, no approved vaccine is registered so far against SARS-CoV-2, but a few candidate vaccines are under trial. A recent study reported the first in human trial of adenovirus type-5 vectored COVID-19 vaccine^24^. Although, some recent studies have proposed peptide-based vaccine targets using the structural glycoproteins^25–31^ but no study has covered all the structural proteins for vaccine designing. With the recent development of integrated bioinformatics and immunoinformatics approaches, vaccine design and its specific application have become rapid and cost-effective. Now a days, the targeted immunogenic peptide can be designed and validated for its efficacy to work on biological systems even before having the vaccine in hand for practical exposure and experiments^32^. However, conventional methods have simultaneously proven effective in vaccine design with some limitations, e.g. sacrificing the whole organism or a large protein residue which unnecessarily increases the antigenic load and probability of allergenicity^33^. We believe that an ideal multi-epitope vaccine can enhance immune response and subsequently lower down the risk of re-infection by enhancing the host immunogenicity. In this perspective, here we applied integrated approaches of vaccine design by mapping B-cell, T-cell and IFN-gamma epitopes present on the four structural proteins of the virus, based on several parameters and then the final proposed vaccine construct was validated using the in-silico immune simulations by injecting the vaccine to monitor the immune response.

## Results

### Target glycoproteins and their antigenicity

We scrutinized target structural protein sequences, i.e. S, M, E and N glycoproteins from the whole-genome of SARS-CoV-2, (GenBank: MN908947.3). Antigenicity prediction for the sequences of structural proteins revealed that E glycoproteins have the highest antigenicity score 0.6025, followed by M, N and S glycoproteins (Table 1).

**Table 1:** Details of target protein and their antigenicity score.

Structural glycoprotein	Protein ID (NCBI)	Size (aa)	Antigenicity score
Surface	QHD43416.1	1273	0.4646
Membrane	QHD43419.1	222	0.5102
Envelop	QHD43418.1	75	0.6025
Nucleocapsid	QHD43423.2	419	0.5059

### Physicochemical and secondary structural properties

Theoretical estimates of isoelectric point (pI) suggested that all the structural proteins were basic and their predicted instability indices were below 40 indicating their stable nature except the N glycoprotein with an instability index of 55.09 (Table S1). The predicted secondary structure characteristics of all the structural proteins showed variable percentage of alpha helix, extended strand, beta turn and random coil (Table S2).

### Three dimensional (3D) structure of target glycoproteins

**Figure 1:**
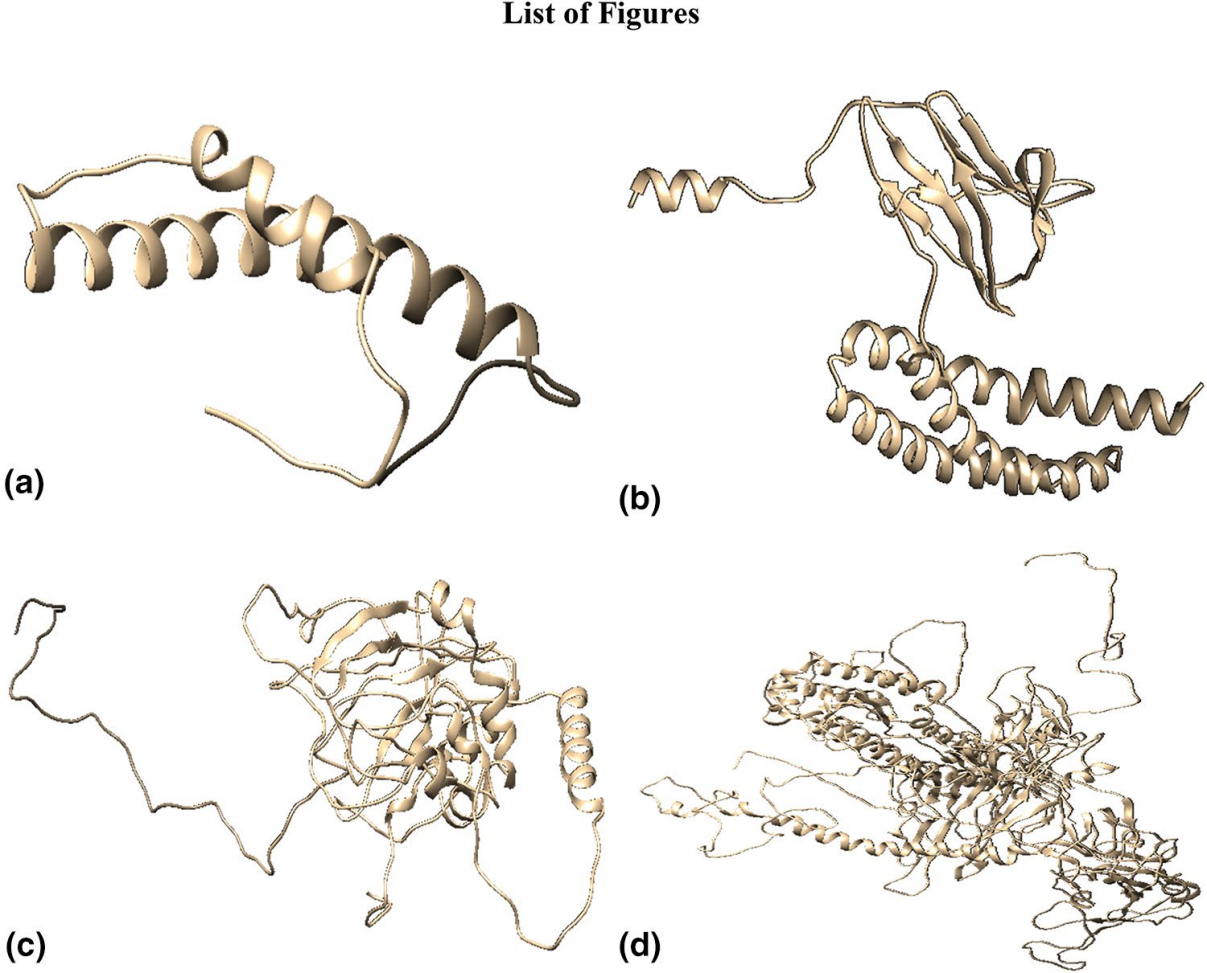
The 3D models of structural proteins of SARS- CoV-2. (**a**) Envelop protein; (**b**) Membrane protein; (**c**) Nucleocapsid protein; (**d**) Surface protein.

The refined finalized 3D structures obtained from Raptor-X showed a very low percentage of outliers and found better than the models generated from I-Tasser and Phyre-2 (Fig. 1). Details of various models of the structural glycoproteins through Ramachandran plot analysis are given in Table S3.

### Prediction of CTL and HTL epitopes

A total of 29 CTL epitopes in E glycoprotein, 77 in N, 340 in S and 89 epitopes in M glycoprotein were predicted with strong binding affinity for multiple alleles. Similarly, 35 HTL epitopes in E glycoprotein, 51 in N, 252 in S and 23 epitopes in M glycoprotein were predicted. All these epitopes were strong binders and predicted percentile rank of ≤ 2.

### Identification of overlapping T cell epitopes

The CTL epitopes overlapping with HTL epitopes were scrutinized and subjected to immunogenicity and allergenicity prediction. In total, 16 CTL overlapping epitopes were predicted in E glycoprotein, 8 in N glycoprotein, 25 in S glycoprotein and 9 epitopes in M glycoprotein (Tables S4–S11). All these selected epitopes were predicted to be non-allergic with high antigenic scores.

### Identification of B-cell epitopes

The identified continuous B cell epitopes present on the surface of the structural proteins were selected based on their antigenic, non-allergic nature coinciding with the CTL and HTL epitopes. With these criteria, five epitopes were obtained in S glycoprotein, three in N and one in M glycoprotein (Table S12). No epitope with such criteria was identified in E glycoprotein. Further, one discontinuous epitope in E glycoprotein, seven in N, nine in S and four discontinuous epitopes were predicted in M glycoprotein (Table S13).

### Identification of IFN-gamma epitopes

The server IFN epitope predicted eight epitopes in E glycoprotein, 39 in M, 66 in N and 281 in S glycoprotein. Based on antigenicity and non-allergenicity, eight epitopes were scrutinized in E glycoprotein, 13 in M, 43 in N and 81 in S glycoprotein. To obtain the highest immunogenicity and reduce the overload of epitopes in vaccine construct, we prioritized one epitope from each glycoprotein with the highest antigenicity score and non-allergenicity (Table S14).

### Characterization of predicted epitopes

#### Conservation analysis, population coverage, and autoimmunity identification

Conservation prediction resulted that all selected T-cell and B-cell epitopes were 100% conserved among the target structural proteins and population coverage prediction of T-cell epitopes showed that five epitopes in E glycoprotein, two in M, two in N and six epitopes in S glycoprotein covered more than 50% of the worldwide population. No selected epitope with > 50% population coverage showed homology with any human protein. Thus, altogether 30 epitopes, 15 each of CTL and HTL, met the above criteria were processed for the interaction analysis with the commonly occurring HLA alleles in the Human population (Table S15).

#### Interaction analysis of epitopes and their HLA alleles

Molecular interaction analysis of each selected CTL and HTL epitopes with their respective HLA alleles resulted in 10 predicted docked complexes. Each complex showed different global energy value. Of the 30 characterized epitopes, those epitopes showing global energy value threshold of − 40 or above were considered as effectively interacting epitopes with the corresponding HLA allele, and thus selected for final vaccine construct (Table 2). This resulted in two CTL and HTL epitopes in E glycoprotein, one in M, two in N and two in S glycoprotein. For an ideal candidate vaccine, the prioritized epitopes included should have a potential affinity towards their respective alleles. Therefore, we selected epitopes showing high interaction with their alleles.

**Table 2:** Final epitopes selected for multi-epitope vaccine construct after docking analysis with respective HLA alleles.

S. no	Protein	HTL	Global energy	CTL	Global energy
1	Envelop	LLFLAFVVFLLVTLA	− 71.44	LLFLAFVVF	− 41.45
2	Envelop	LAFVVFLLVTLAILT	− 64.39	FLLVTLAIL	− 43.31
3	Membrane	TLACFVLAAVYRINW	− 53.12	TLACFVLAA	− 54.85
4	Nucleocapsid	GDAALALLLLDRLNQ	− 87.95	GDAALALLL	− 40.49
5	Nucleocapsid	AQFAPSASAFFGMSR	− 58.74	AQFAPSASA	− 43.29
6	Surface	IPFAMQMAYRFNGIG	− 46.33	FAMQMAYRF	− 41.37
7	Surface	FVFLVLLPLVSSQCV	− 74.92	FVFLVLLPL	− 55.15

#### Construction of final multi-epitope vaccine

**Figure 2:**
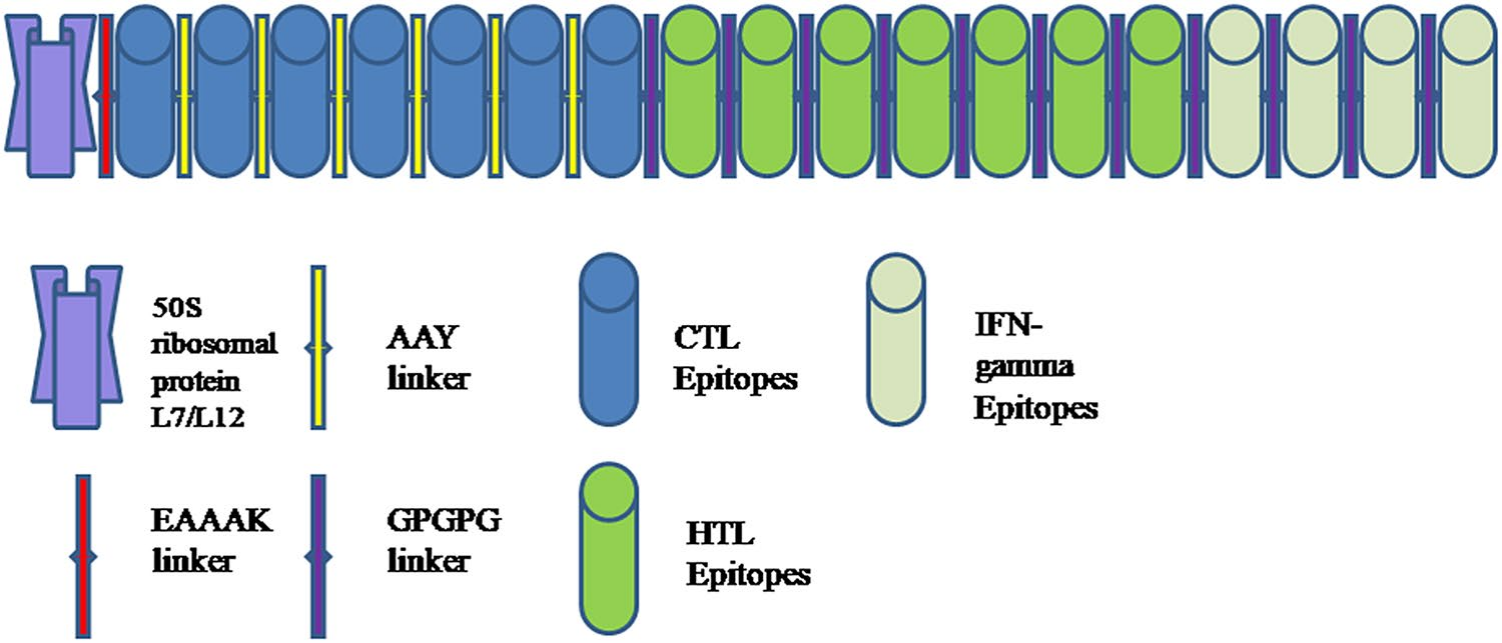
Schematic diagram of final vaccine construct.

Finally, we selected 18 epitopes, i.e. seven CTL, seven HTL and four IFN-gamma for the development of the multi-epitope vaccine (Figure S1). The adjuvant, i.e. 50S ribosomal protein L7/L12 was coupled by the EAAAK linker with CTL epitope and subsequently, AAY linker was used to couple CTL epitopes and GPGPG linker was used to couple HTL and IFN-gamma epitopes (Fig. 2). The final multi-epitope vaccine construct was composed of 430 amino acid residues which were then validated for antigenic, allergenic and physicochemical properties.

#### Evaluation of antigenicity, allergenicity, physicochemical properties and secondary structure of the vaccine construct

The multi-epitope vaccine construct found to be immunogenic (Ag score- 0.627), non-allergic with 45.131 KDa molecular weight. The prediction suggested that vaccine construct was stable and basic with instability index 23.46 and theoretical pI 9.01. The half-life of the vaccine in mammalian reticulocytes (in vitro) was 30 h, while it was about 20 h in yeast (in-vivo) and 10 h in *E. coli* (in vivo). The predicted secondary structure of the vaccine construct consisted of 42.33% alpha-helix, 22.09% extended strand, 4.42% beta-turn and 31.16% random coil.

#### Modelling, refinement, and validation of vaccine construct

**Figure 3:**
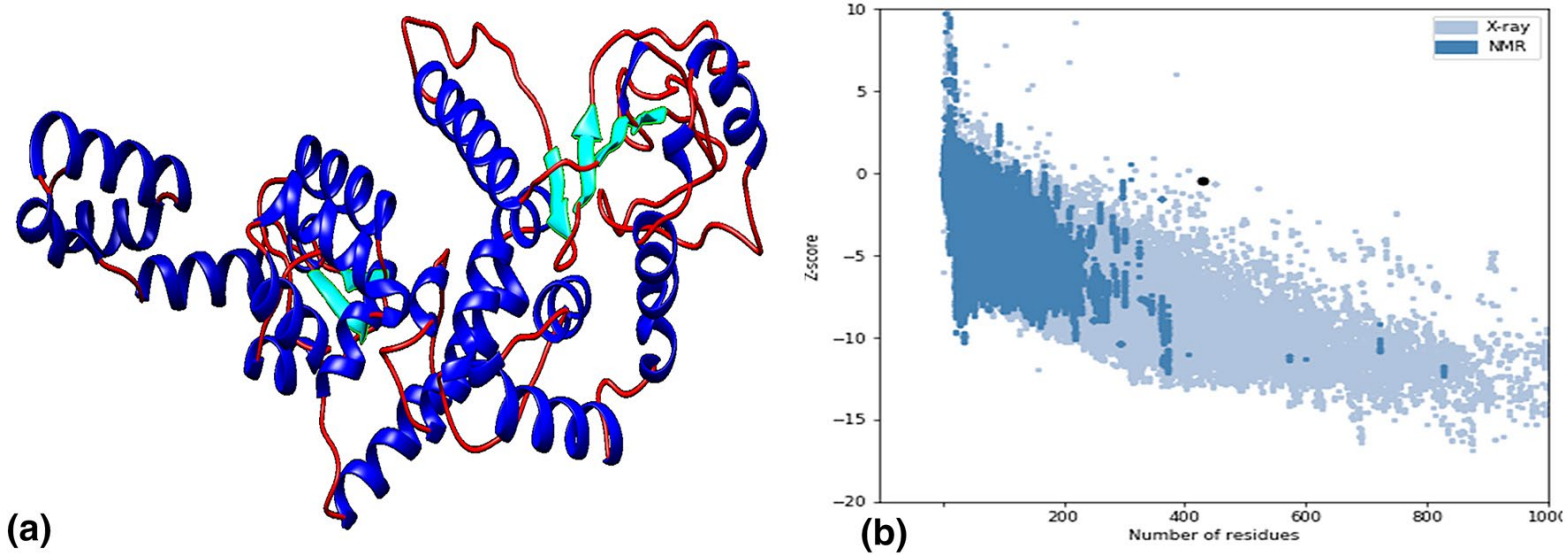
(**a**) 3 dimensional structure of final vaccine construct, (**b**) ProSA validation of predicted structure with Z score of − 0.47.

Of the top 10 predicted models, the model with the Z-score of 7.61 with template 1dd3A was selected for improvement. The ProSA-web tool assessed the overall and local quality of the refined model with a Z-score of − 0.47, found close to the range of native protein of similar size (Fig. 3). Further, the Ramachandran plot analysis revealed 92% of residues in the favoured region, 6.1% in the allowed region and 1.9% outliers.

#### Prediction of continuous and discontinuous B-cell epitopes

We found four continuous epitopes,- AKILKEKYGLD, EILDKSKEKTSFD, LKESKDLV and VPKHLKKGLSKEEAESLKKQLEEV on the surface of predicted vaccine following prediction made by BCpred 2.0 server (Figure S2), while Ellipro server predicted six discontinuous epitopes with a score higher than 0.8 (Figure S3; Table S16).

#### Molecular docking of vaccine construct with TLR3 and TLR4 receptor

**Figure 4:**
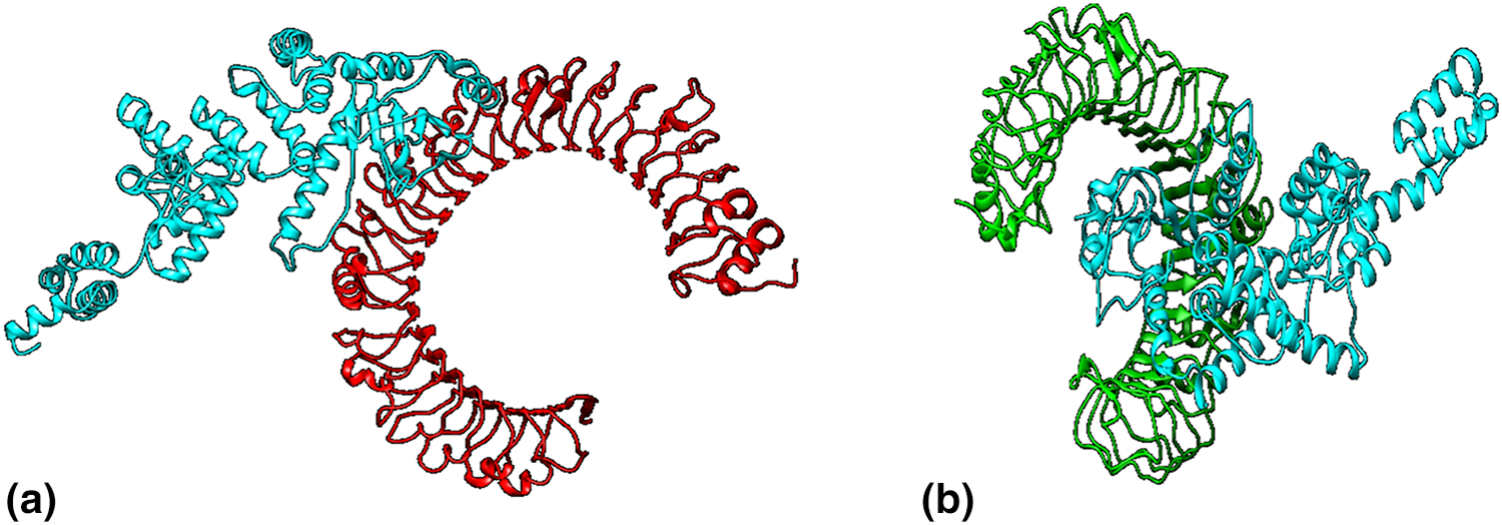
The interaction pattern of designed vaccine with TLR3 and TLR4. (**a**) Vaccine (Cyan) docked with receptor TLR3 (Red). (**b**) Vaccine (Cyan) docked with receptor TLR4 (Green).

Cluspro v.2 predicted 30 models each of vaccine receptor TLR3 complex and TLR4 complex with their corresponding cluster scores (Table S17–S18). Among these models, the model 2 in TLR3 complex and model 1 in TLR4 complex was selected as a best-docked complex with the lowest energy score of − 1199.1 with 46 members (TLR3) and lowest energy score of − 1229.9 with 79 members (TLR4). This signifies potential molecular interaction between predicted vaccine construct with TLR3 and TLR 4 receptors (Fig. 4).

#### In-silico cloning and vaccine optimization

**Figure 5:**
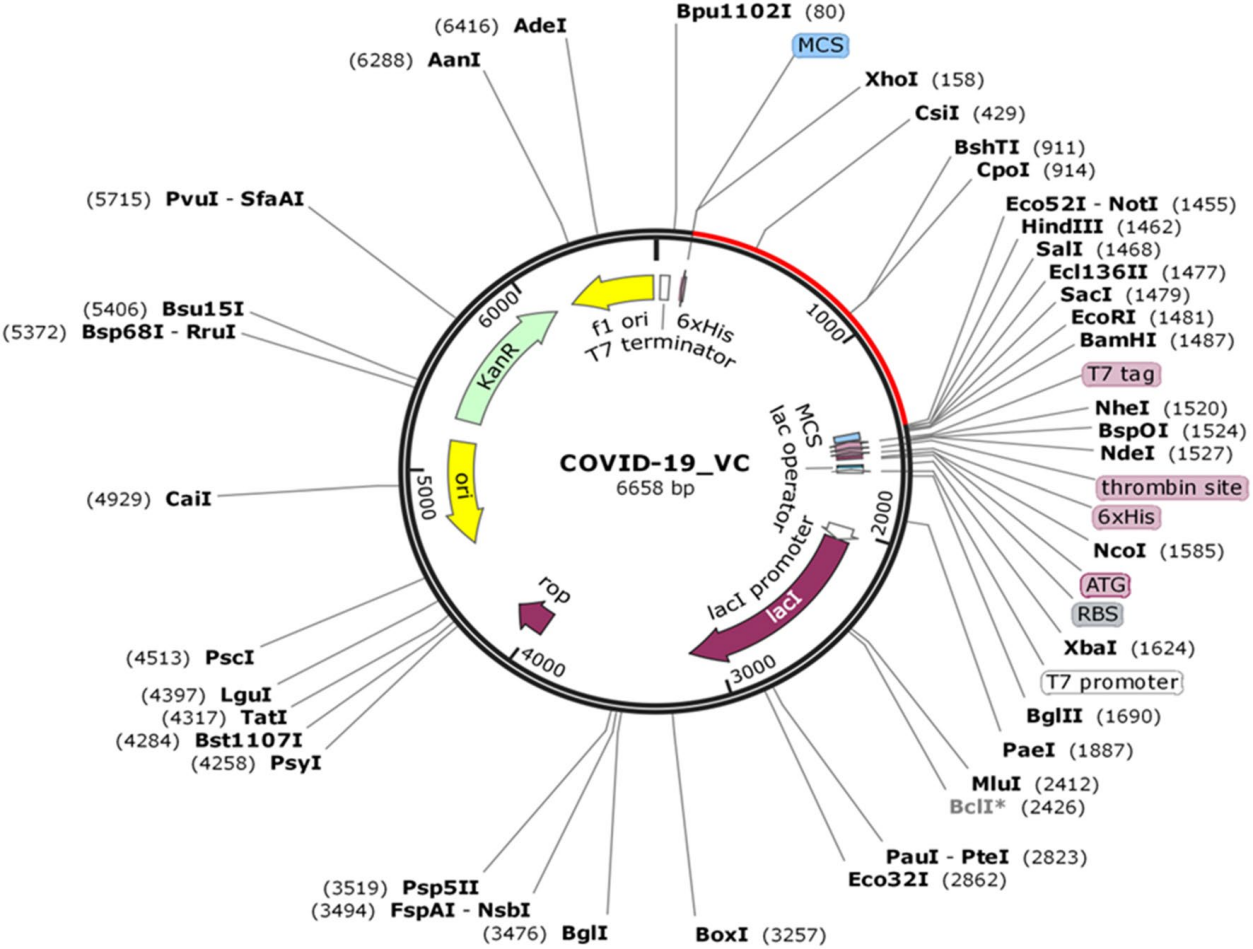
In silico cloning of vaccine. The segment represented in red is the multi-epitope vaccine insert in pET-28(+) expression vector.

The Java Codon Adaptation Tool optimized the codon usage of the vaccine and produced an optimized codon sequence of 1290 nucleotides. The Codon Adaptation Index (CAI) of the optimized sequences was 0.95, and the GC content was 54.41%, which ensured the potential, expression of vaccine construct in the host cell, *E. coli*. Later on, the restriction sequence of Xho I and Not I restriction enzymes were added to N and C terminal of adapted codon sequence. Further, the adapted sequence was also cloned in pET-28(+) vector using SnapGene tool (Fig. 5).

#### Immune simulations of vaccine construct

**Figure 6:**
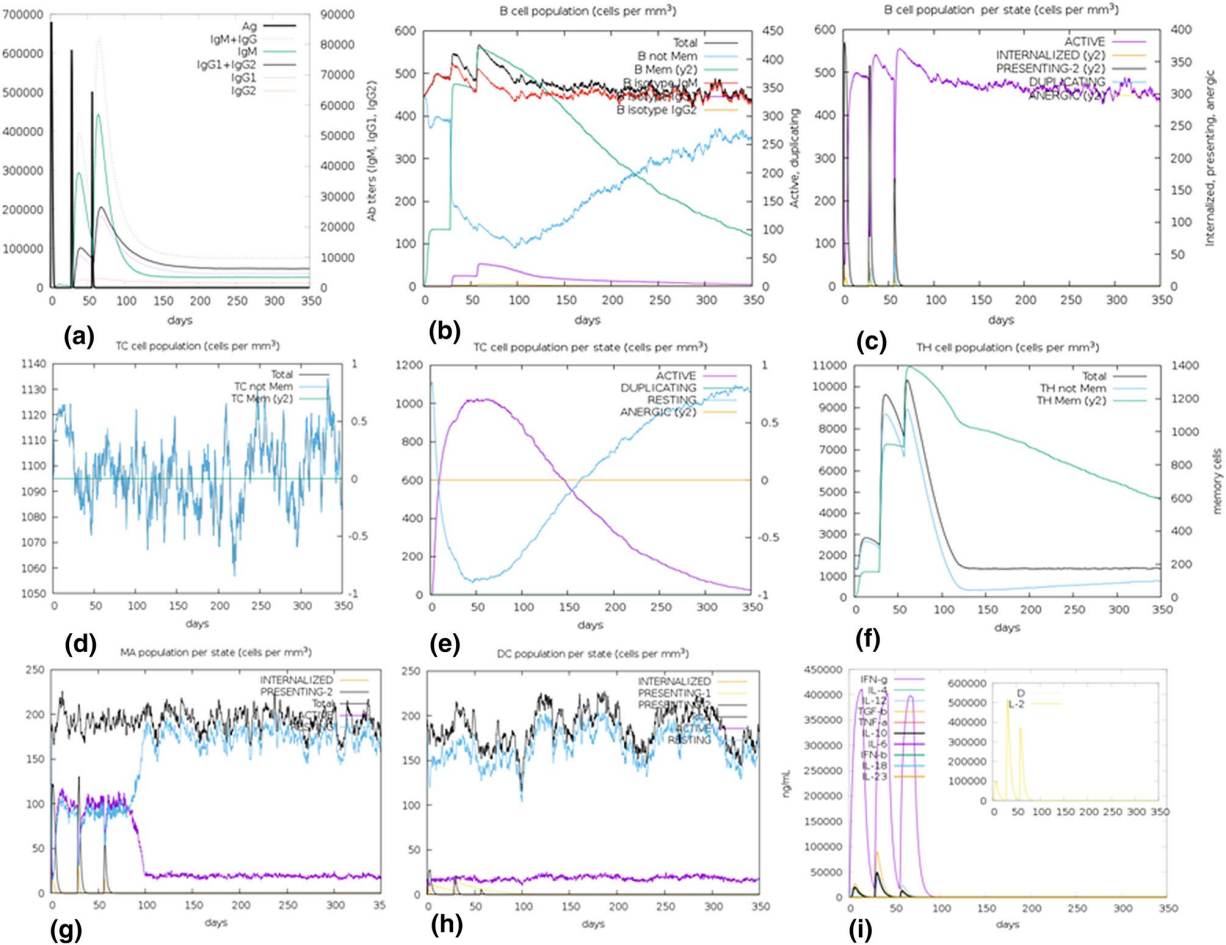
In silico simulation of immune response using vaccine as an antigen after subsequent three injections. (**a**) Antigen and Immunoglobins. (**b**) B-cell population. (**c**) B-cell population per state. (**d**) Cytotoxic T-cell population. (**e**) Cytotoxic T-cell population per state. (**f**) Helper T-cell population. (**g**) Macrophages population per state. (**h**) Dendritic cell population per state. (**i**) Cytokine production.

The simulations result suggested that administration of the vaccine by three injections was good enough to potentially induce various immunoglobulins. The primary response appeared by the increase in the level of IgM and the secondary response was characterized by an increased level of IgM + IgG, IgG1 + IgG2, IgG1, IgG2, and B-cell populations. On the subsequent exposure of three injections of the vaccine, a decrease in the level of antigens was observed. Both T cell populations, i.e. CTL and HTL, developed increased response corresponding to the memory cells signifying the immunogenicity of T cell epitopes included in the vaccine construct. Increased activity of macrophages was also observed at each exposure, whereas the activity of NK cells was observed consistently throughout the period. A significant increase in the level of IFN-gamma, IL-10, IL-23, and IL-12 was also found at subsequent exposure (Fig. 6). After the repeated exposure of vaccine construct through 12 injections, on a regular interval, the level of antigen attained a similar peak (figure S4-a), but showed a remarkable increase in the level of IgM + IgG, IgG1 + IgG2. A surge in the memory corresponding to B cell and T cells were observed throughout the exposure while the level of IFN-gamma was consistently high from first to last exposure (Figure S4). This signifies that the vaccine generated a robust immune response in case of short exposure and immunity increases even on subsequent repeated exposure.

## Discussion

The recent outbreak of SARS-CoV-2 in Wuhan city of China raised several questions regarding the susceptibility of humans against this novel pathogen. As predicted earlier about the re-emergence of unique variants of SARS-CoV^34^, SARS-CoV-2 has spread more widely in a short period as compared to the previous variants. This escalated rate of transmission initiated a pursuit for vaccine development against SARS-CoV-2, which already caused 646,641 deaths as on July 27th 2020^2^. With strict and comprehensive measures, vaccine development and its application could play a key role in eliminating the virus from the human population or to restrain the community spread. Thus several efforts are being made to address the challenge that appeared in the current scenario with appreciable advancements in the understanding of virus biology and its etiology^35^. The lack of knowledge regarding the response of the immune system against viral infection is one of the major limitations in the path of vaccine development for SARS-CoV-2.

This study is a novel attempt in describing the potential immunogenic target using the all four structural glycoproteins of the virus and proposes a novel multi-epitope vaccine construct, by providing detailed insights in the initial phase of vaccine development. Since, immunity against any antigen is prominently dependent on, how it gets recognized by B cells and T cells. We identified epitopes corresponding to B cells and T cells in each structural protein so that both innate and adaptive immunity can be induced with the exposure of vaccine construct. Further, IFN-gamma epitopes were identified which are equally effective in immune regulatory, antiviral and antitumor activities. The final vaccine construct was designed with due consideration of the overlapping T-cell epitopes and IFN-gamma epitopes to increase the possibilities of immune recognition ability. The epitopes were prioritized following various parameters, e.g. nature of epitopes being antigenic, non-allergic, conservation efficiency among the target proteins, their affinity for multiple alleles, homology search with any of the human proteins, their vast coverage on human populations and effective molecular interaction with their respective HLA alleles. The highest antigenicity score for E glycoprotein showed the most potent candidate to generate immune response. Considering the functional role of E glycoprotein in the life history traits of the virus, Schoeman and Fielding^23^ recently reported that those CoVs lacking E protein make promising candidate vaccine. The designed vaccine of 430 amino acid residues with the molecular weight of 45.131 KDa, also found well fit within the defined range (40–50 KDa) of average molecular weight of a multi-epitope vaccine^36^. The instability index and theoretical pI also suggested that the vaccine is stable, basic in nature and the estimated half-life suggested that the recognized peptide does not possess a short half-life and would remain viable for a span adequate to generate a potent immune response. Vaccine model refinement and validation indicated that the quality of the predicted model was good as more than 90% residues were in the favoured region^37^. The B-cell epitopes were predicted on the surface of the vaccine, which showed its effectiveness to be recognized by the B-cells. Since TLR 3 and TLR 4 have proven recognition capability in both SARS-CoV and MERS-CoV^38,39^ and considering a similar genome organization of SARS-CoV-2 like SARS-CoV, the molecular interaction of vaccine through docking analysis suggested that candidate vaccine has significant affinity to TLR 3 and TLR 4 to act as a sensor for recognizing molecular patterns of pathogen and initiating immune response. Thus, the vaccine TLR complex is capable of generating an effective innate and adaptive immune response against SARS-CoV-2. Further, Codon adaptation improved the expression of the candidate vaccine in *E. coli*. strain K12 with significant codon adaptation index and GC content indicating elevated expression level. After successful cloning of the candidate vaccine in the pET-28(+) vector, the simulation-based generated immune response, suggested that the primary and secondary immune response will be coherent with the actually expected response. Subsequent exposure for three injections was found adequate in generating effective immunogenic response. The consistently high level of IFN-gamma also supported the activation of innate immunity. However, repeated exposure of the vaccine by administrating 12 periodic injections suggested an obvious more stringent immune response. All the immunoinformatics approaches demonstrated that vaccine designed against SARS-CoV-2 yielded promising results by reducing antigen growth and inducing immune response.

Further, to seek the convenient proof and reliability of the immunoinformatic approaches used in designing the potential candidate vaccine, we compared the predicted epitopes with the experimentally determined SARS-CoV-derived B cell and T cell epitopes^30^. We observed thirteen epitopes i.e. six T-cell nucleocapsid epitopes, one T-cell surface epitope, two linear B cell epitopes and four discontinuous B-cell epitopes, were either identical and or overlapping with Ahmed et al.^30^ with high antigenicity and allergenicity scores (Table S19). Since, we have considered all four structural proteins to screen T-cell, B-cell and IFN gamma epitopes for designing the potential candidate vaccine, the present study provide a comprehensive and better insights for vaccine design against SARS-CoV-2.

Without following the standard operating procedures and guidelines issued time to time by WHO, it is difficult to curb coronavirus pandemic given the situation when no vaccine is available. However, a few therapeutics have been recently tested against SARS-CoV-2^5,40^, but none of them showed complete effectiveness to cure the disease. In this study, a vaccinomics approach was carried out to design a multi-epitope vaccine against SARS-CoV-2 using several in-silico and immunoinformatic methods. We propose that the designed vaccine has all the potential to induce both the innate and adaptive immune systems and can neutralize the SARS-CoV-2. Therefore, we appeal for an experimental validation of the designed vaccine must be undertaken by the professionals/clinicians for reaching a conclusion for its safety, efficacy and success.

### Methodology

#### Retrieval of SARS-CoV-2 protein sequences and antigenicity prediction

Sequences of all the four structural glycoproteins of SARS-CoV-2 were retrieved from NCBI (GenBank: MN908947.3). We predicted antigenicity from each protein using the online server VaxiJen v2.0^41^ and proteins that showed antigenicity ≥ 0.4 in the virus category were subjected for further analysis.

#### Prediction of physicochemical and secondary structural properties of the target proteins

The physicochemical properties of target proteins were examined using Expasy Protpram online server^42^ and the conformational states, e.g. Helix, sheet, turn and coil was predicted using the online Secondary structure analysis tool (SOPMA)^43^ with default parameters.

#### 3D homology modelling and validation

The 3D structure of target proteins was modelled using three homology modelling tools, i.e. I-Tasser, Raptor-X and Phyre2^44–46^ and post processing to reduce any distortion in the modelled structure was done using the Galaxy refine server^47^. The modelled structures were subjected to RAMPAGE server for Ramachandran plot analysis for quality check and validation^48^ and visualized using Chimera 1.10.1 visualization system^49^.

### Prediction of T-cell epitopes

#### Cytotoxic T-cell (CTL) epitopes

Nine residues long CTL epitopes recognized by HLA class-I supertypes, i.e. A1, A2, A3, A24, A26, B7, B8, B27, B39, B44, B58, B62 were predicted using NetCTL.1.2 server^50^ considering the default values of weight on C terminal cleavage, weight on TAP transport efficiency and the threshold for epitope identification. Further, another set of CTL epitopes recognized by HLA class I alleles like A*02:02, B*07:02, C*04:01, E*01:01, etc. were identified by consensus method of Immune Epitope Database (IEDB) tool^51^. The identified epitopes were scrutinized based on the consensus score, i.e. ≤ 2 and only those epitopes were considered as strong binders and selected for further analysis, which were predicted by more than one allele by both the methods.

#### Helper T-cell (HTL) epitopes

Fifteen residue long HTL epitopes recognized by HLA Class II DRB1 alleles were predicted using Net MHC II pan 3.2 servers^52^. The threshold for strong binder and weak binder were set as default. Further another set of HTL epitopes of 15 residues length recognized by HLA DR alleles were identified using the consensus method as implemented in the IEDB server^51^. The epitopes with a percentile rank of ≤ 2 and predicted by more than one allele by both methods were considered as strong binders and scrutinized for further analysis.

#### Identification of overlapping T-cell epitopes

Considering the fact that epitopes with an affinity for multiple HLA alleles tend to induce relatively more immune response in the host cell, we scrutinized overlapping epitopes with affinity to both HLA class I and class II alleles for immunogenicity and allergen prediction using VaxiJen v2.0 server^41^ and AllerTop v.2.0^53^ tool with an immunogenic threshold of 0.4. These epitopes were assumed to show high potential to activate both CTL and HTL cells.

#### Identification of continuous and discontinuous B-cell epitopes

B-cell epitopes are short amino acid sequences recognized by the surface-bound receptors of B-lymphocyte. Their identification plays a major role in vaccine design, and thus two types-continuous and discontinuous B-cell epitopes were predicted over the corresponding structural protein sequences. The continuous B-cell epitopes, present on the surface of proteins, were predicted using BCpred 2.0^54^ server and the discontinuous epitopes which were reasonably large were predicted using Ellipro server^55^. The prediction parameters like Minimum score and Maximum distance (Anstrom) were set as 0.8 and 6, respectively.

#### Identification of IFN-gamma epitopes

Since IFN-gamma is the signature cytokine of both the innate and adaptive immune system, therefore epitopes with potency to induce IFN-gamma could boost the immunogenic capacity of any vaccine. Thus, IFN-gamma epitopes were predicted for each structural protein by the IFN epitope server^56^.

### Characterization of predicted epitopes

#### Conservation analysis

Predicted T-cell and B-cell epitopes were submitted to the IEDB conservancy analysis tool^57^ to identify the degree of conservancy in the structural protein sequences, and the epitopes with 100% conservancy were selected for further analysis.

#### Population coverage and autoimmunity identification

The immunogenic response to human population towards the selected overlapping CTL and HTL epitopes against their respective HLA genotype frequencies was predicted using IEDB population coverage analysis tool^58^. Those epitopes exhibiting 50% or more population coverage were scrutinized. To reduce the probability of autoimmunity, all the selected epitopes were subjected to Blastp search analysis against the Human proteome. Epitopes showing similarity to any human protein were excluded from further analysis.

#### Interaction analysis of epitopes and their HLA alleles

The sequence of scrutinized epitopes was submitted to an online server PEPFOLD 3^59^ for 3D structure modelling. The structure of the most common HLA alleles, i.e. HLA-DRB1 *01:01 (HLA class II) and HLA-A* 02:01 (HLA class I) in human population were retrieved from Protein data bank (PDB) with a PDB ID of 2G9H and 1QEW. Any ligand associated with the HLA allele’s structure was removed, and energy-minimization was carried out. After that, the modelled epitopes were docked with the corresponding HLA allele using a web server Patchdock^60^, to study the interaction pattern of receptor and ligand. The parameters like clustering RMSD value and the complex type was kept as 0.4 and default. The result obtained from Patchdock was forwarded to the Firedock server^61^ for the refinement of the best 10 models. Based on Global energy value, the complex with the lowest global energy was scrutinized, and the corresponding CTL and HTL epitopes were selected for the final vaccine construct.

#### Construction and quality control of final multi-epitope vaccine

For the construction of a multi-epitope vaccine against SARS-CoV-2, we followed Chauhan et al.^33^ with some modifications. The epitopes were prioritized for selection based on meeting the following criteria: (1) epitopes must be immunogenic, non-allergic and possessing overlapping affinity to both HLA class I and class II alleles; (2) epitopes must be capable of activating both CTL and HTL cells and must have a minimum 50% of the population coverage; (3) epitopes should not be overlapping with any human gene, and the predicted B-Cell epitopes should be present on the surface of the target protein. Based on meeting these criteria, the HTL, CTL and IFN-gamma epitopes were selected in the final construct of the multi-epitope vaccine. The HTL and IFN-gamma epitopes were linked by GPGPG linkers, whereas the CTL epitopes were linked by AAY linkers^33^. An adjuvant, 50S ribosomal protein L7/L12 (Locus RL7_MYCTU) with an NCBI accession no: P9WHE3 was added to the N terminal to enhance the immunogenicity of the vaccine construct.

#### Antigenicity, allergenicity and physicochemical properties of the vaccine

The antigenicity of the proposed vaccine was predicted using VaxiJen v2.0 server^41^, and allergenicity was predicted using Allertop v.2.0 prediction tool^53^. The physicochemical property of the designed vaccine was predicted by submitting the final sequence of the vaccine to the Expasy Protpram online server^42^.

#### Prediction of secondary structure

The secondary structure of the vaccine construct was predicted using the SOPMA tool^43^. With the input of vaccine sequence and output width 70, the parameters like the number of conformational states, similarity threshold, and window width were set as 4 (Helix, Sheet, Turn, Coil), 8 and 17.

#### Modelling, refinement, and validation of vaccine construct

The final model of the vaccine construct was prepared using a homology modelling tool SPARKS-X^62^. Among the top 10 predicted models, the model with the highest Z-score was selected and subjected for refinement using the Galaxy refine server^47^. Then after, we utilized ProSA-webtool^63^ to assess the overall and local quality of the predicted model based on the Z-score. If the Z-score lies outside the range of native protein of similar size, a chance of error increases in the predicted structure. Further to check the overall quality of the refined structure of vaccine, Ramachandran plot analysis was carried out using the RAMPAGE server^48^. The final model of the vaccine construct was visualized using Chimera 1.10.1 visualization system^49^.

#### Prediction of continuous and discontinuous epitopes

The continuous and discontinuous B-cell epitopes in the vaccine construct were predicted using BCpred 2.0^54^ and Ellipro server^55^, respectively. For the effective design of vaccine, the vaccine construct should posses B-cell epitopes for potential enhancement of immunogenic reactions.

#### Molecular docking of vaccine construct with TLR3 and TLR4 receptor

The 3D structures of human TLR3 and TLR4 were retrieved from protein data bank (PDB ID: 2A0Z and 2z63). Any ligand attached to the retrieved structures was removed, and the receptor was subjected for model refinement using Galaxy refine server^47^. Molecular docking analysis was performed using the Cluspro v.2 protein–protein docking server^64^ to analyze the interaction pattern of vaccine with TLR3 and TLR4. The server provides cluster scores based on rigid docking by sampling billions of conformation, pairwise RMSD energy minimization. Based on the lowest energy weight score and members, the final vaccine-TLR3 complex and TLR4 complex model was selected and visualized using Chimera 1.10.1 visualization system^49^.

#### *In-silico* cloning and vaccine optimization

The efficiency of the multi-epitope vaccine construct in cloning and expression is of the utmost importance for vaccine design. The final multi-epitope vaccine construct was submitted to the Java Codon Adaptation Tool (JCat)^65^, where codon optimization was performed in *E. coli* strain K12. In addition to this, parameters like Avoid rho-independent transcription terminators, Avoid prokaryotic ribosome binding sites, Avoid Cleavage Sites of Restriction Enzymes were selected for the final submission. The output of the JCat tool provided CAI value and GC content of the optimized sequence. Ideally, the CAI value should be greater than 0.8 or nearer to 1^66^, and the GC content should be in a range of 30 to 70%^67^ to ensure that the vaccine has a potential translation, stability and transcription efficiency. Further, an *in-silico* cloning of a multi-epitope vaccine construct in *E. coli* pET–28(+) vector was performed using the SnapGene 4.2 tool^68^.

#### Immune simulations of vaccine construct

To characterize the real-life immunogenic profiles and immune response of the multi-epitope vaccine, C-ImmSim server^69^ was utilized. Position-specific scoring matrix (PSSM) and machine learning are the two methods, based on which C-ImmSim predicts immune epitopes and immune interactions. It simultaneously simulates three different anatomical regions of mammals, i.e. Bone marrow, Thymus and tertiary lymphatic organs. To conduct the immune simulation, three injections at an interval of four weeks were administered with 1000 vaccine molecules per injection. The parameters like random seed, simulation volume, and simulation step were kept as 12,345, 10 µl and 1050, respectively. Several studies suggested that the minimum interval between two injections should be kept four weeks and therefore, the time steps followed for three injections were 1, 84 and 168, where each time step is equal to 8 h of real-life^70^. Since, several patients worldwide got recurrent infection of SARS-CoV-2, 12 consecutive injections were administered four weeks apart for the assessment of after-effects of vaccine exposure on SARS-CoV-2.

## Supplementary information

Supplementary figures.

Supplementary tables.

## Data Availability

All relevant data is included in the manuscript.
