# Supplementary material for: Designing a multi-epitope peptide based vaccine against SARS-CoV-2
Source: Sci Rep. 2020 Oct 1;10:16219. doi: 10.1038/s41598-020-73371-y (PMC7530768; doi:10.1038/s41598-020-73371-y)
Supplement: Supplementary file 1 — Supplementary figures. [file 41598_2020_73371_MOESM1_ESM.docx]

Short title: **A multi-epitope peptide based vaccine against SARS-CoV-2**

Running title: **Designing a multi-epitope peptide based vaccine against SARS-CoV-2**

**Abhishek Singh, Mukesh Thakur, Lalit Kumar Sharma and Kailash Chandra**

**List of supplementary figures**


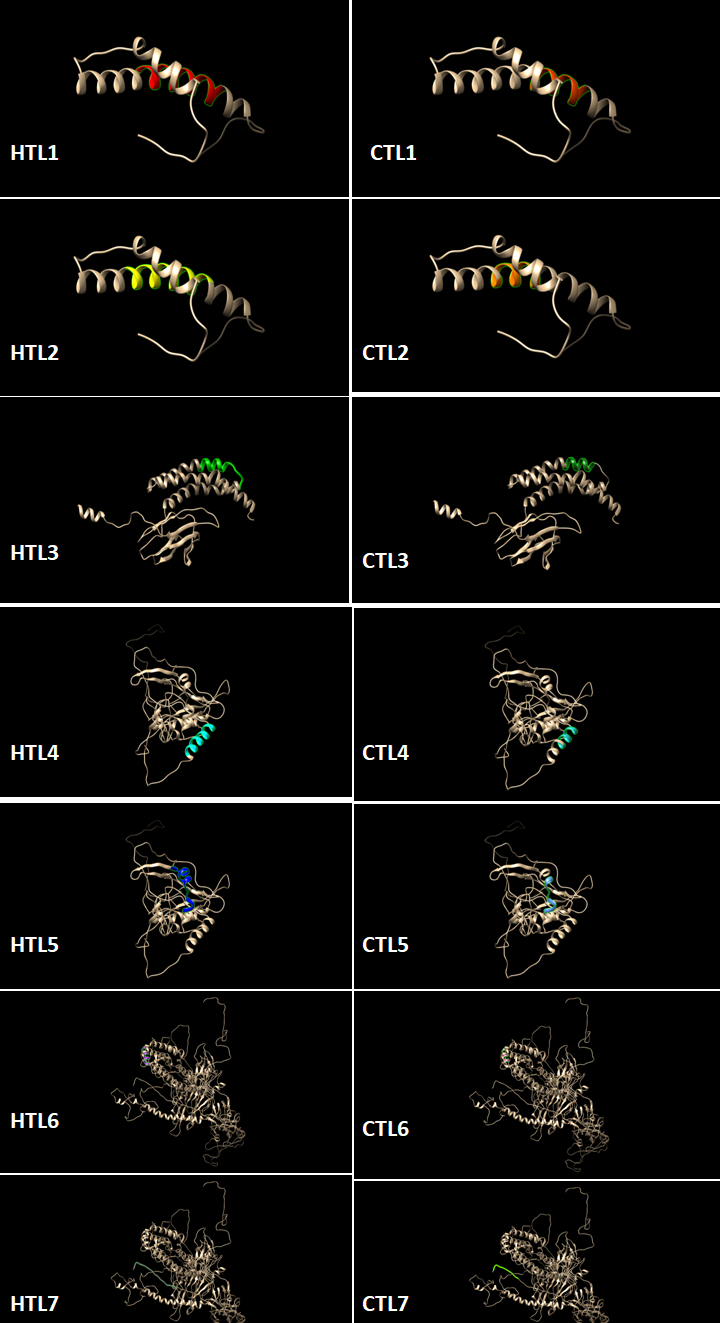


**Envelop**

**Envelop**

**Envelop**

**Envelop**

**Membrane**

**Membrane**

**Nucleocapsid**

**Nucleocapsid**

**Nucleocapsid**

**Nucleocapsid**

**Surface**

**Surface**

**Surface**

**Surface**

Figure S1. CTL and HTL epitopes (coloured) visualized on their respective structural glycoproteins


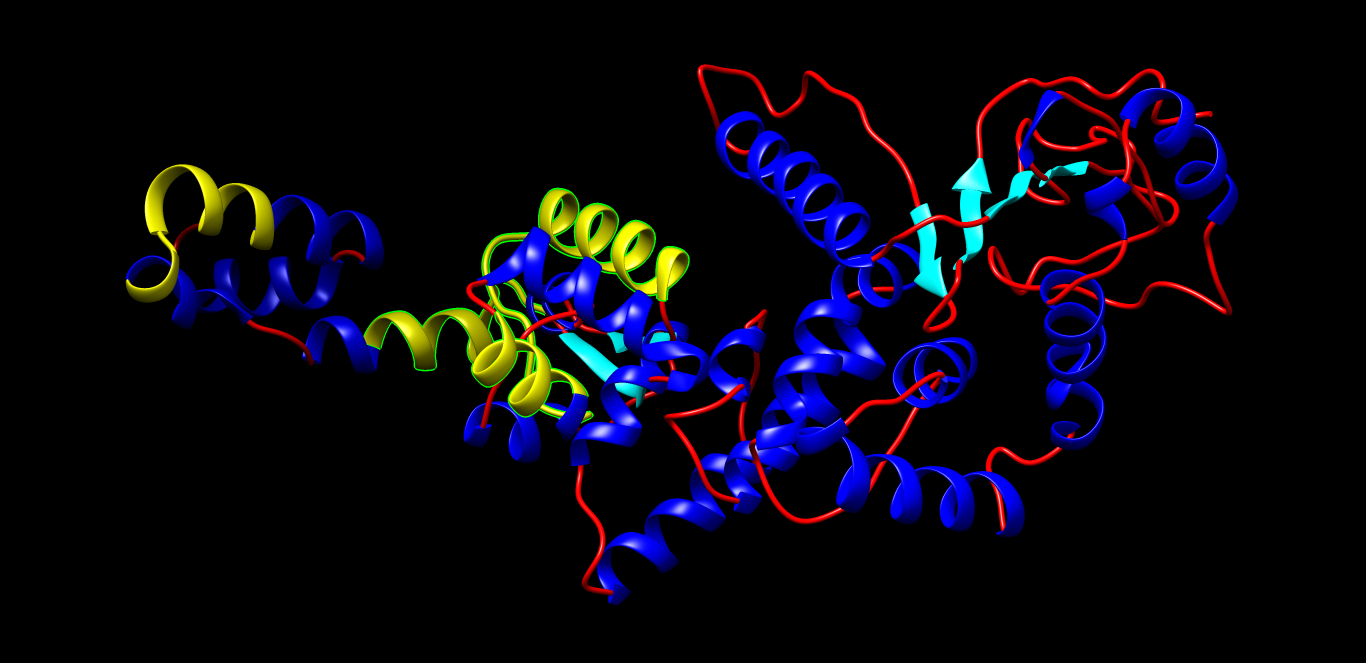


Figure S2. Predicted continuous epitopes ( Yellow) on the surface of designed vaccine

Figure S3 Predicted discontinuous epitopes on the surface of designed vaccine


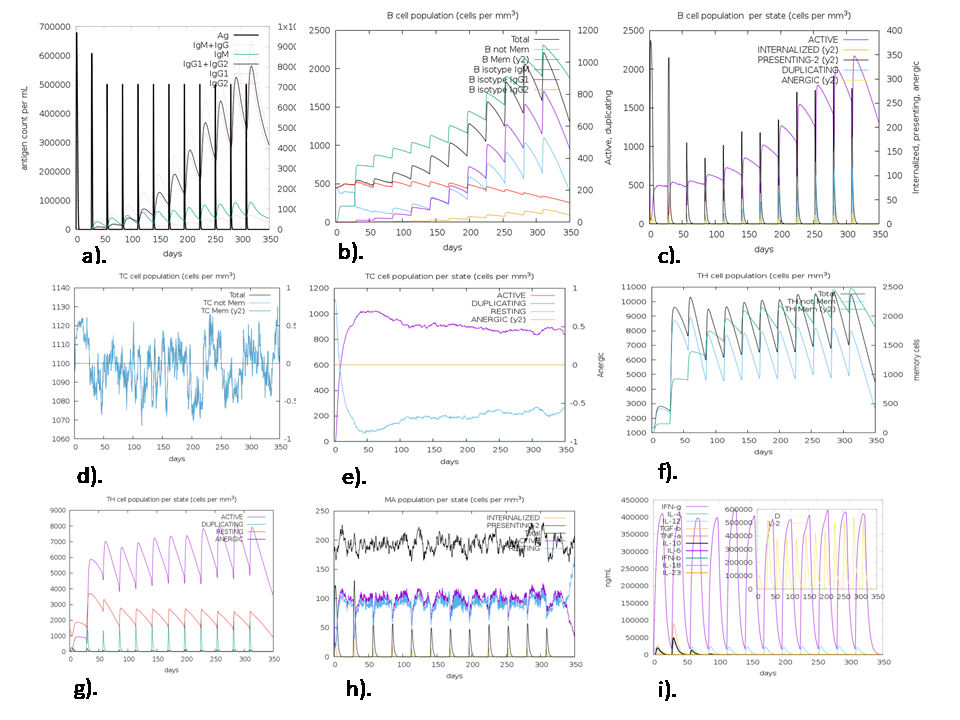


Figure S4. In silico simulation of immune response using vaccine as an antigen after subsequent 12 injections. a). Antigen and Immunoglobins. b). B-cell population. c). B-cell population per state. d). Cytotoxic T-cell population. e). Cytotoxic T-cell population per state. f). Helper T-cell population. g). Helper T-cell population per state. h). Macrophages population per state. i). Cytokine production.
