# Supplementary material for: Designing a multi-epitope peptide based vaccine against SARS-CoV-2
Source: Sci Rep. 2020 Oct 1;10:16219. doi: 10.1038/s41598-020-73371-y (PMC7530768; doi:10.1038/s41598-020-73371-y)
Supplement: Supplementary file 2 — Supplementary tables. [file 41598_2020_73371_MOESM2_ESM.doc]

Short title: **A multi-epitope peptide based vaccine against SARS-CoV-2**

Running title: **Designing a multi-epitope peptide based vaccine against SARS-CoV-2**

**Abhishek Singh^1,2^, Mukesh Thakur^1^, Lalit Kumar Sharma^1^ and Kailash Chandra^1^**

**List of supplementary tables**

Table S1 Physicochemical properties of structural glycoproteins

| **Structural Glycoprotein** | **Molecular weight** | **Theoretical pI** | **Instability index** | **Aliphatic index** | **GRAVY** |
| --- | --- | --- | --- | --- | --- |
| Surface | 141178.47 | 6.24 | 33.01 | 84.67 | -0.079 |
| Membrane | 25146.62 | 9.51 | 39.14 | 120.86 | 0.446 |
| Envelop | 8365.04 | 8.57 | 38.68 | 144.00 | 1.128 |
| Nucleocapsid | 45625.70 | 10.07 | 55.09 | 52.53 | -0.971 |

Table S2. Secondary structural properties of target proteins

| **Structural Glycoprotein** | **Alpha Helix** | **3_10_ Helix** | **pI helix** | **Beta Bridge** | **Extended Strand** | **Beta Turn** | **Bend Region** | **Random Coil** | **Ambiguous States** | **Other states** |
| --- | --- | --- | --- | --- | --- | --- | --- | --- | --- | --- |
| Surface | 28.59% | 0% | 0% | 0% | 23.25% | 3.38% | 0% | 44.78% | 0% | 0% |
| Membrane | 34.68% | 0% | 0% | 0% | 21.17% | 6.76% | 0% | 37.39% | 0% | 0% |
| Envelop | 44% | 0% | 0% | 0% | 26.67% | 9.33% | 0% | 20% | 0% | 0% |
| Nucleocapsid | 21.24% | 0% | 0% | 0% | 16.71% | 6.92% | 0% | 55.13% | 0% | 0% |

Table S3. Structural details of the modelled proteins

| **Structural Glycoprotein** | **Ramachandran Plot Analysis** | | | | | | | | |
| --- | --- | --- | --- | --- | --- | --- | --- | --- | --- |
|  | **I Tasser** | | | **Raptor-X** | | | **Phyre2** | | |
|  | **Favoured region** | **Allowed region** | **Outlier region** | **Favoured region** | **Allowed region** | **Outlier region** | **Favoured region** | **Allowed region** | **Outlier region** |
| Surface | 77.9% | 16.5% | 5.6% | 93.0% | 5.9% | 1.1% | 91.5% | 7.4% | 1.1% |
| Membrane | 95.5% | 4.1% | 0.5% | 97.3% | 2.7% | 0.0% | 100.0% | 0.0% | 0.0% |
| Envelop | 91.8% | 8.2% | 0.0% | 97.3% | 2.7% | 0.0% | 96.4% | 0.0% | 3.6% |
| Nucleocapsid | 82.7% | 14.6% | 2.6% | 92.7% | 7.0% | 0.3% | 92.3% | 6.4% | 1.3% |

Table S4 Overlapping CTL epitopes in Envelop protein

| **Envelop Protein (CTL)** | | | | |
| --- | --- | --- | --- | --- |
| **Position** | **CTL** | **Antigenicity** | **Allergenicity** | **Supertypes/HLA Alleles** |
| 20 | FLAFVVFLL | 0.5308 | Non Allergen | A2,A24,B8,B39,HLA-A*02:01,HLA-A*02:06,HLA-A*68:02,HLA-B*39:01,HLA-B*38:01,HLA-B*15:02,HLA-E*01:03 |
| 26 | FLLVTLAIL | 0.9645 | Non Allergen | A2,B8,B39,HLA-A*02:01,HLA-A*02:06,HLA-B*08:01,HLA-B*15:02,HLA-B*39:01 |
| 23 | FVVFLLVTL | 0.7403 | Non Allergen | A2,A26,B8,HLA-A*02:06,HLA-A*25:01,HLA-A*02:01,HLA-A*68:02,HLA-B*39:01 |
| 21 | LAFVVFLLV | 0.7976 | Non Allergen | A2,HLA-A*02:06,HLA-A*68:02,HLA-B*51:01,HLA-B*58:01,HLA-C*12:03 |
| 31 | LAILTALRL | 0.8872 | Non Allergen | B58,HLA-B*14:02,HLA-B*51:01,HLA-B*53:01,HLA-C*15:02 |
| 19 | LFLAFVVFL | 0.4568 | Non Allergen | A24,HLA-A*23:01,HLA-C*04:01 |
| 18 | LLFLAFVVF | 0.8144 | Non Allergen | B8,B62,HLA-A*23:01,HLA-B*15:01,HLA-A*32:01,HLA-B*35:01 |
| 15 | NSVLLFLAF | 0.4134 | Non Allergen | B62,HLA-A*23:01,HLA-B*15:01,HLA-B*35:01,HLA-B*18:01,HLA-B*46:01,HLA-B*53:01 |
| 61 | RVKNLNSSR | 0.8998 | Non Allergen | A3,HLA-A*31:01,HLA-A*30:01,HLA-A*68:01 |
| 55 | SFYVYSRVK | 0.8251 | Non Allergen | A3,HLA-A*03:01,HLA-A*30:01,HLA-C*14:02 |
| 16 | SVLLFLAFV | 0.4765 | Non Allergen | A2,A26,HLA-A*02:06,HLA-A*02:01,HLA-A*68:02 |
| 30 | TLAILTALR | 0.7223 | Non Allergen | A3,HLA-A*03:01,HLA-A*68:01,HLA-A*31:01 |
| 25 | VFLLVTLAI | 0.8134 | Non Allergen | A24,HLA-A*23:01,HLA-A*24:02,HLA-C*14:02 |
| 17 | VLLFLAFVV | 0.5677 | Non Allergen | A2,HLA-A*02:01,HLA-A*02:06,HLA-E*01:03 |
| 29 | VTLAILTAL | 0.614 | Non Allergen | A2,HLA-A*02:06,HLA-B*48:01 |
| 57 | YVYSRVKNL | 0.702 | Non Allergen | A2,B8,B39,B62,HLA-A*25:01,HLA-B*08:01,HLA-C*03:03,HLA-C*06:02,HLA-C*07:02,HLA-C*14:02 |

Table S5 Overlapping HTL epitopes in Envelop protein

| **Envelop Protein (HTL)** | | | | |
| --- | --- | --- | --- | --- |
| **Position** | **HTL** | **Antigenicity** | **Allergenicity** | **Supertypes/HLA Alleles** |
| 18 | LLFLAFVVFLLVTLA | 0.8122 | Non Allergen | HLA-DRB1*01:02,HLA-DRB1*04:23,HLA-DRB1*04:04,HLA-DRB1*04:08,HLA-DRB1*04:10,HLA-DRB1*07:03,HLA-DRB1*04:05,HLA-DRB1*08:13,HLA-DRB1*15:02 |
| 21 | LAFVVFLLVTLAILT | 0.8229 | Non Allergen | HLA-DRB1*01:02,HLA-DRB1*04:01,HLA-DRB1*04:23,HLA-DRB1*04:08,HLA-DRB1*04:10,HLA-DRB1*04:21,HLA-DRB1*04:26,HLA-DRB1*07:03,HLA-DRB1*04:05,HLA-DRB1*11:04,HLA-DRB1*11:06,HLA-DRB1*08:13,HLA-DRB1*11:28,HLA-DRB1*13:05,HLA-DRB1*15:02,HLA-DRB1*13:11 |
| 21 | LAFVVFLLVTLAILT | 0.8229 | Non Allergen | HLA-DRB1*01:02,HLA-DRB1*04:01,HLA-DRB1*04:23,HLA-DRB1*04:08,HLA-DRB1*04:10,HLA-DRB1*04:21,HLA-DRB1*04:26,HLA-DRB1*07:03,HLA-DRB1*04:05,HLA-DRB1*11:04,HLA-DRB1*11:06,HLA-DRB1*08:13,HLA-DRB1*11:28,HLA-DRB1*13:05,HLA-DRB1*15:02,HLA-DRB1*13:11 |
| 21 | LAFVVFLLVTLAILT | 0.8229 | Non Allergen | HLA-DRB1*01:02,HLA-DRB1*04:01,HLA-DRB1*04:23,HLA-DRB1*04:08,HLA-DRB1*04:10,HLA-DRB1*04:21,HLA-DRB1*04:26,HLA-DRB1*07:03,HLA-DRB1*04:05,HLA-DRB1*11:04,HLA-DRB1*11:06,HLA-DRB1*08:13,HLA-DRB1*11:28,HLA-DRB1*13:05,HLA-DRB1*15:02,HLA-DRB1*13:11 |
| 29 | VTLAILTALRLCAYC | 0.8599 | Non Allergen | DRB1_0102,DRB1_0104,DRB1_0120,DRB1_0126,DRB1_0410,DRB1_0412,DRB1_0418,DRB1_0442,DRB1_0453,DRB1_0458,DRB1_0810,DRB1_1113,DRB1_1118,DRB1_1134,DRB1_1167,DRB1_1201,DRB1_1203,DRB1_1205,DRB1_1206,DRB1_1207,DRB1_1208,DRB1_1210,DRB1_1211,DRB1_1212,DRB1_1214,DRB1_1216,DRB1_1217,DRB1_1219,DRB1_1306,DRB1_1344,DRB1_1377,DRB1_1417,DRB1_1431,DRB1_1432,DRB1_1435,DRB1_1452,DRB1_1455,DRB1_1465,DRB1_1478,DRB1_1521,DRB1_0828,DRB1_1002,HLA-DRB1*01:01,HLA-DRB1*01:02 |
| 18 | LLFLAFVVFLLVTLA | 0.8122 | Non Allergen | HLA-DRB1*01:02,HLA-DRB1*04:23,HLA-DRB1*04:04,HLA-DRB1*04:08,HLA-DRB1*04:10,HLA-DRB1*07:03,HLA-DRB1*04:05,HLA-DRB1*08:13,HLA-DRB1*15:02 |
| 18 | LLFLAFVVFLLVTLA | 0.8122 | Non Allergen | HLA-DRB1*01:02,HLA-DRB1*04:23,HLA-DRB1*04:04,HLA-DRB1*04:08,HLA-DRB1*04:10,HLA-DRB1*07:03,HLA-DRB1*04:05,HLA-DRB1*08:13,HLA-DRB1*15:02 |
| 15 | NSVLLFLAFVVFLLV | 0.422 | Non Allergen | HLA-DRB1*07:03,HLA-DRB1*15:01,HLA-DRB1*15:06,HLA-DRB1*15:02 |
| 55 | SFYVYSRVKNLNSSR | 0.6291 | Non Allergen | DRB1_0401,DRB1_0405,DRB1_0408,DRB1_0409,DRB1_0416,DRB1_0419,DRB1_0421,DRB1_0424,DRB1_0426,DRB1_0429,DRB1_0430,DRB1_0433,DRB1_0434,DRB1_0435,DRB1_0438,DRB1_0445,DRB1_0447,DRB1_0448,DRB1_0454,DRB1_0457,DRB1_0461,DRB1_0462,DRB1_0463,DRB1_0464,DRB1_0475,DRB1_0476,DRB1_0477,DRB1_0480,DRB1_0483,DRB1_0484,DRB1_0489,DRB1_0706,DRB1_0801,DRB1_0805,DRB1_0808,DRB1_0811,DRB1_1109,DRB1_1110,DRB1_1112,DRB1_1115,DRB1_1124,DRB1_1127,DRB1_1128,DRB1_1130,DRB1_1129,DRB1_1132,DRB1_1133,DRB1_1137,DRB1_1139,DRB1_1149,DRB1_1151,DRB1_1153,DRB1_1161,DRB1_1162,DRB1_1166,DRB1_1169,DRB1_1174,DRB1_1175,DRB1_1180,DRB1_1181,DRB1_1187,DRB1_1190,DRB1_1191,DRB1_1194,DRB1_1195,DRB1_1196,DRB1_1305,DRB1_1307,DRB1_1314,DRB1_1326,DRB1_1347,DRB1_1349,DRB1_1350,DRB1_1355,DRB1_1360,DRB1_1362,DRB1_1388,DRB1_1422,DRB1_1425,DRB1_1427,DRB1_1446,DRB1_1453,DRB1_1515,DRB1_1601,DRB1_1603,DRB1_1604,DRB1_1608,DRB1_1609,DRB1_0816,DRB1_0824,DRB1_0826,DRB1_0839,DRB1_1101,HLA-DRB1*04:01,HLA-DRB1*07:03,HLA-DRB1*04:05,HLA-DRB1*08:02,HLA-DRB1*09:01 |
| 52 | VKPSFYVYSRVKNLN | 1.2319 | Non Allergen | DRB1_0706,DRB1_1515,DRB1_1601,DRB1_1603,DRB1_1608,DRB1_1609,DRB1_1504,HLA-DRB1*07:03 |
| 16 | SVLLFLAFVVFLLVT | 0.5446 | Non Allergen | HLA-DRB1*07:03,HLA-DRB1*15:01,HLA-DRB1*15:06,HLA-DRB1*15:02 |
| 29 | VTLAILTALRLCAYC | 0.8599 | Non Allergen | DRB1_0102,DRB1_0104,DRB1_0120,DRB1_0126,DRB1_0410,DRB1_0412,DRB1_0418,DRB1_0442,DRB1_0453,DRB1_0458,DRB1_0810,DRB1_1113,DRB1_1118,DRB1_1134,DRB1_1167,DRB1_1201,DRB1_1203,DRB1_1205,DRB1_1206,DRB1_1207,DRB1_1208,DRB1_1210,DRB1_1211,DRB1_1212,DRB1_1214,DRB1_1216,DRB1_1217,DRB1_1219,DRB1_1306,DRB1_1344,DRB1_1377,DRB1_1417,DRB1_1431,DRB1_1432,DRB1_1435,DRB1_1452,DRB1_1455,DRB1_1465,DRB1_1478,DRB1_1521,DRB1_0828,DRB1_1002,HLA-DRB1*01:01,HLA-DRB1*01:02 |
| 21 | LAFVVFLLVTLAILT | 0.8229 | Non Allergen | HLA-DRB1*01:02,HLA-DRB1*04:01,HLA-DRB1*04:23,HLA-DRB1*04:08,HLA-DRB1*04:10,HLA-DRB1*04:21,HLA-DRB1*04:26,HLA-DRB1*07:03,HLA-DRB1*04:05,HLA-DRB1*11:04,HLA-DRB1*11:06,HLA-DRB1*08:13,HLA-DRB1*11:28,HLA-DRB1*13:05,HLA-DRB1*15:02,HLA-DRB1*13:11 |
| 17 | VLLFLAFVVFLLVTL | 0.6386 | Non Allergen | HLA-DRB1*07:03,HLA-DRB1*15:02,HLA-DRB1*15:01,HLA-DRB1*15:06 |
| 29 | VTLAILTALRLCAYC | 0.8599 | Non Allergen | DRB1_0102,DRB1_0104,DRB1_0120,DRB1_0126,DRB1_0410,DRB1_0412,DRB1_0418,DRB1_0442,DRB1_0453,DRB1_0458,DRB1_0810,DRB1_1113,DRB1_1118,DRB1_1134,DRB1_1167,DRB1_1201,DRB1_1203,DRB1_1205,DRB1_1206,DRB1_1207,DRB1_1208,DRB1_1210,DRB1_1211,DRB1_1212,DRB1_1214,DRB1_1216,DRB1_1217,DRB1_1219,DRB1_1306,DRB1_1344,DRB1_1377,DRB1_1417,DRB1_1431,DRB1_1432,DRB1_1435,DRB1_1452,DRB1_1455,DRB1_1465,DRB1_1478,DRB1_1521,DRB1_0828,DRB1_1002,HLA-DRB1*01:01,HLA-DRB1*01:02 |
| 52 | VKPSFYVYSRVKNLN | 1.2319 | Non Allergen | DRB1_0706,DRB1_1515,DRB1_1601,DRB1_1603,DRB1_1608,DRB1_1609,DRB1_1504,HLA-DRB1*07:03 |

Table S6 Overlapping CTL epitopes in Nucleocapsid protein

| **Nucleocapsid Protein (CTL)** | | | | |
| --- | --- | --- | --- | --- |
| **Position** | **CTL** | **Antigenicity** | **Allergenicity** | **Supertypes/HLA Alleles** |
| 305 | AQFAPSASA | 0.7468 | Non Allergen | B62,HLA-A*02:06,HLA-B*15:01,HLA-B*48:01 |
| 215 | GDAALALLL | 0.4529 | Non Allergen | B44,HLA-B*40:02,HLA-B*48:01,HLA-E*01:01 |
| 316 | GMSRIGMEV | 0.6287 | Non Allergen | A2,HLA-A*02:01 |
| 219 | LALLLLDRL | 0.5933 | Non Allergen | HLA-B*14:02,HLA-B*51:01 |
| 306 | QFAPSASAF | 0.5495 | Non Allergen | A24,B62,HLA-A*23:01,HLA-A*24:02,HLA-A*29:02,HLA-B*15:02,HLA-B*35:01,HLA-C*04:01,HLA-B*46:01 |
| 105 | SPRWYFYYL | 0.734 | Non Allergen | B7,B8,HLA-B*08:01,HLA-B*07:02,HLA-B*35:03,HLA-B*51:01,HLA-B*53:01 |
| 329 | TWLTYTGAI | 0.5439 | Non Allergen | A24,HLA-A*24:02,HLA-A*23:01,HLA-C*14:02 |
| 109 | YFYYLGTGP | 1.2234 | Non Allergen | HLA-C*04:01,HLA-C*14:02 |

Table S7 Overlapping HTL epitopes in Nucleocapsid protein

| **Nucleocapsid Protein (HTL)** | | | | |
| --- | --- | --- | --- | --- |
| **Position** | **HTL** | **Antigenicity** | **Allergenicity** | **Supertypes/HLA Alleles** |
| 305 | AQFAPSASAFFGMSR | **0.5266** | Non Allergen | HLA-DRB1*09:01,DRB1_0901,DRB1_0902,DRB1_0904,DRB1_0907,DRB1_0909 |
| 215 | GDAALALLLLDRLNQ | **0.4458** | Non Allergen | HLA-DRB1*11:04,HLA-DRB1*11:06,HLA-DRB1*11:02,HLA-DRB1*08:17,HLA-DRB1*08:06,HLA-DRB1*08:04,HLA DRB1*11:21,HLA-DRB1*13:04,HLA-DRB1*11:28,HLA-DRB1*13:05,HLA-DRB1*11:14,HLA-DRB1*13:11,HLA-DRB1*13:21,HLA-DRB1*13:07,HLA-DRB1*13:22,HLA-DRB1*13:23,HLA-DRB4*01:01 |
| 311 | ASAFFGMSRIGMEVT | **0.862** | Non Allergen | HLA-DRB1*11:28,HLA-DRB1*13:05,HLA-DRB1*13:21,DRB1_1130 |
| 219 | LALLLLDRLNQLESK | **0.7357** | Non Allergen | HLA-DRB1*11:04,HLA-DRB1*11:06,HLA-DRB1*11:02,HLA-DRB1*08:17,HLA-DRB1*08:06,HLA-DRB1*08:04,HLA-DRB1*11:21,HLA-DRB1*13:04,HLA-DRB1*11:28,HLA-DRB1*13:05,HLA-DRB1*11:14,HLA-DRB1*13:11,HLA-DRB1*13:21,HLA-DRB1*13:07,HLA-DRB1*13:22,HLA DRB1*13:23,DRB1_0301,DRB1_0302,DRB1_0303,DRB1_0304,DRB1_0305,DRB1_0306,DRB1_0307,DRB1_0308,DRB1_0310,DRB1_0311,DRB1_0313,DRB1_0314,DRB1_0315,DRB1_0318,DRB1_0319,DRB1_0320,DRB1_0321,DRB1_0322,DRB1_0323,DRB1_0324,DRB1_0325,DRB1_0326,DRB1_0328,DRB1_0329,DRB1_0330,DRB1_0331,DRB1_0332,DRB1_0333,DRB1_0334,DRB1_0336,DRB1_0337,DRB1_0338,DRB1_0339,DRB1_0340,DRB1_0341,DRB1_0342,DRB1_0343,DRB1_0344,DRB1_0345,DRB1_0346,DRB1_0347,DRB1_0348,DRB1_0349,DRB1_0350,DRB1_0351,DRB1_0352,DRB1_0353,DRB1_0354,DRB1_0355,DRB1_0422,DRB1_1107,DRB1_1404,DRB1_1413,DRB1_1428,DRB1_1438,DRB1_1447,DRB1_1450,DRB1_1461,DRB1_1471,DRB1_1476,DRB1_1479,DRB1_1493,DRB1_1525 |
| 305 | AQFAPSASAFFGMSR | **0.5266** | Non Allergen | HLA-DRB1*09:01,DRB1_0901,DRB1_0902,DRB1_0904,DRB1_0907,DRB1_0909 |
| 105 | SPRWYFYYLGTGPEA | **0.8767** | Non Allergen | HLA-DRB1*04:05,HLA-DRB1*13:21,DRB1_0424,DRB1_0480,DRB1_0908 |
| 329 | TWLTYTGAIKLDDKD | **1.2416** | Non Allergen | HLA-DRB1*07:01,HLA-DRB1*07:03,DRB1_0701,DRB1_0703,DRB1_0705,DRB1_0706,DRB1_0707,DRB1_0708,DRB1_0709,DRB1_0712,DRB1_0713,DRB1_0714,DRB1_0715,DRB1_0716,DRB1_0717,DRB1_0719 |
| 105 | SPRWYFYYLGTGPEA | **0.8767** | Non Allergen | HLA-DRB1*04:05,HLA-DRB1*13:21,DRB1_0424,DRB1_0480,DRB1_0908 |

Table S8 Overlapping CTL epitopes in Surface protein

| **Surface Protein (CTL)** | | | | | |
| --- | --- | --- | --- | --- | --- |
| **Position** | **HTL** | **Antigenicity** | **Allergenicity** | **Supertypes/HLA Alleles** | |
| 892 | **AALQIPFAM** | **0.775** | Non Allergen | B7,B58,HLA-B*35:01,HLA-B*46:01,HLA-C*03:03 | |
| 890 | **AGAALQIPF** | **0.486** | Non Allergen | B62,HLA-B*15:01 | |
| 1226 | **AIVMVTIML** | **0.755** | Non Allergen | A2,B7 |  |
| 903 | **AYRFNGIGV** | **1.3** | Non Allergen | HLA-A*30:01,HLA-C*14:02 | |
| 898 | **FAMQMAYRF** | **1.028** | Non Allergen | B58,B62,HLA-A*23:01,HLA-A*24:02,HLA-A*29:02,HLA-B*08:01,HLA-B*15:01,HLA-A*32:01,HLA-B*35:01,HLA-B*46:01,HLA-B*53:01,HLA-B*58:01,HLA-B*51:01,HLA-B*57:01 | |
| 515 | **FELLHAPAT** | **0.541** | Non Allergen | HLA-B*40:02,HLA-B*18:01 | |
| 329 | **FPNITNLCP** | **1.622** | Non Allergen | HLA-B*35:03,HLA-B*53:01 | |
| 2 | **FVFLVLLPL** | **0.86** | Non Allergen | A2,A26,B8,B62,HLA-A*02:06,HLA-A*25:01,HLA-A*02:01,HLA-A*26:01,HLA-A*68:02,HLA-B*39:01,HLA-B*35:03,HLA-B*46:01,HLA-B*48:01 | |
| 1059 | **GVVFLHVTY** | **1.41** | Non Allergen | B62,HLA-A*29:02,HLA-A*32:01 | |
| 1221 | **IAGLIAIVM** | **0.472** | Non Allergen | HLA-B*35:01,HLA-B*46:01 | |
| 1225 | **IAIVMVTIM** | **1.134** | Non Allergen | B58,HLA-B*35:01,HLA-B*46:01,HLA-B*51:01,HLA-B*58:01,HLA-B*53:01,HLA-C*03:03,HLA-B*57:01 | |
| 202 | **KIYSKHTPI** | **0.746** | Non Allergen | A2,B8,HLA-A*32:01,HLA-B*08:01,HLA-B*48:01,HLA-C*15:02 | |
| 1224 | **LIAIVMVTI** | **1.112** | Non Allergen | A2,HLA-A*32:01,HLA-B*51:01 | |
| 821 | **LLFNKVTLA** | **0.615** | Non Allergen | A2,HLA-A*02:01 | |
| 897 | **PFAMQMAYR** | **1.332** | Non Allergen | HLA-A*31:01,HLA-C*04:01 | |
| 628 | **QLTPTWRVY** | **1.212** | Non Allergen | A1,B62,HLA-A*29:02 | |
| 901 | **QMAYRFNGI** | **0.68** | Non Allergen | HLA-B*08:01,HLA-A*32:01 | |
| 328 | **RFPNITNLC** | **1.217** | Non Allergen | HLA-A*24:02,HLA-C*04:01 | |
| 827 | **TLADAGFIK** | **0.578** | Non Allergen | A3,HLA-A*11:01,HLA-A*03:01,HLA-A*68:01,HLA-E*01:03 | |
| 109 | **TLDSKTQSL** | **1.069** | Non Allergen | A2,B39,HLA-A*02:01,HLA-B*08:01,HLA-B*14:02,HLA-C*08:02,HLA-E*01:01 | |
| 630 | **TPTWRVYST** | **0.461** | Non Allergen | B7,HLA-B*07:02 | |
| 3 | **VFLVLLPLV** | **0.465** | Non Allergen | A24,HLA-A*23:01,HLA-C*04:01 | |
| 327 | **VRFPNITNL** | **1.114** | Non Allergen | B27,B39,HLA-B*14:02,HLA-B*27:05,HLA-B*38:01,HLA-C*06:02,HLA-C*07:02,HLA-C*07:01 | |
| 1060 | **VVFLHVTYV** | **1.512** | Non Allergen | A2,HLA-A*02:06,HLA-A*02:01,HLA-A*68:02,HLA-C*06:02,HLA-C*07:01 | |
| 886 | **WTFGAGAAL** | **0.492** | Non Allergen | A26,B62,HLA-A*26:01,HLA-A*68:02,HLA-B*07:02,HLA-B*15:02,HLA-B*15:02,HLA-B*39:01,HLA-B*38:01,HLA-B*48:01,HLA-B*46:01 | |

Table S9 Overlapping HTL epitopes in Surface protein

| **Surface Protein (HTL)** | | | | |
| --- | --- | --- | --- | --- |
| **Position** | **HTL** | **Antigenicity** | **Allergenicity** | **Supertypes/HLA Alleles** |
| 892 | AALQIPFAMQMAYRF | **0.911** | Non Allergen | HLA-DRB4*01:01,DRB1_0102,DRB1_0103,DRB1_0104,DRB1_0106,DRB1_0109,DRB1_0110,DRB1_0115,DRB1_0116,DRB1_0120,DRB1_0123,DRB1_0126 |
| 885 | GWTFGAGAALQIPFA | **0.467** | Non Allergen | HLA-DRB1*01:01,HLA-DRB1*01:01,HLA-DRB1*09:01,DRB1_0101,DRB1_0105,DRB1_0107,DRB1_0108,DRB1_0110,DRB1_0112,DRB1_0119,DRB1_0121,DRB1_0122,DRB1_0124,DRB1_0125,DRB1_0127,DRB1_0128,DRB1_0129,DRB1_0130,DRB1_0131,DRB1_0132 |
| 1225 | IAIVMVTIMLCCMTS | **0.744** | Non Allergen | HLA-DRB1*03:06,HLA-DRB1*03:07,HLA-DRB1*03:08,HLA-DRB1*03:05,HLA-DRB1*03:06,HLA-DRB1*03:07,HLA-DRB1*03:08,HLA-DRB1*03:05,HLA-DRB1*11:04,HLA-DRB1*11:06,HLA-DRB1*08:04,HLA-DRB1*08:13,HLA-DRB1*11:07,HLA-DRB1*11:28,HLA-DRB1*13:05,HLA-DRB1*13:11,HLA-DRB1*13:07,HLA-DRB1*13:21,HLA-DRB1*15:06 |
| 900 | MQMAYRFNGIGVTQN | **1.303** | Non Allergen | HLA-DRB1*13:01,HLA-DRB1*13:27,HLA-DRB1*13:28 |
| 896 | IPFAMQMAYRFNGIG | **1.283** | Non Allergen | HLA-DRB1*13:01,HLA-DRB1*13:27,HLA-DRB1*13:29,DRB1_0101,DRB1_0102,DRB1_0103,DRB1_0104,DRB1_0105+D815,DRB1_0106,DRB1_0107,DRB1_0108,DRB1_0109,DRB1_0110,DRB1_0112,DRB1_0113,DRB1_0114,DRB1_0115,DRB1_0118,DRB1_0119,DRB1_0120,DRB1_0122,DRB1_0123,DRB1_0125,DRB1_0126,DRB1_0127,DRB1_0128,DRB1_0129,DRB1_0130,DRB1_0131,DRB1_0132 |
| 511 | VVLSFELLHAPATVC | **0.862** | Non Allergen | HLA-DRB1*01:01,HLA-DRB1*01:01,HLA-DRB1*15:06,DRB1_0101,DRB1_0105,DRB1_0107,DRB1_0108,DRB1_0109,DRB1_0110,DRB1_0111,DRB1_0112,DRB1_0113,DRB1_0114,DRB1_0117,DRB1_0118,DRB1_0119,DRB1_0121,DRB1_0122,DRB1_0124,DRB1_0125,DRB1_0127,DRB1_0128,DRB1_0129,DRB1_0130,DRB1_0131,DRB1_0132 |
| 325 | SIVRFPNITNLCPFG | **0.79** | Non Allergen | HLA-DRB1*15:06,HLA-DRB1*15:02 |
| 2 | FVFLVLLPLVSSQCV | **0.719** | Non Allergen | HLA-DRB1*01:01,HLA-DRB1*01:01,HLA-DRB1*11:01,HLA-DRB1*11:02,HLA-DRB1*11:04,HLA-DRB1*11:06,HLA-DRB1*08:17,HLA-DRB1*11:28,HLA-DRB1*13:05,HLA-DRB1*11:21,HLA-DRB1*13:01,HLA-DRB1*11:14,HLA-DRB1*13:21,HLA-DRB1*15:02,HLA-DRB1*13:07,HLA-DRB1*13:22,HLA-DRB1*13:11,HLA-DRB1*13:27,HLA-DRB1*13:28,HLA-DRB1*13:23 |
| 1059 | GVVFLHVTYVPAQEK | **1.104** | Non Allergen | HLA-DRB1*01:02,HLA-DRB1*04:02,HLA-DRB1*01:02,HLA-DRB1*04:02 |
| 1221 | IAGLIAIVMVTIMLC | **0.434** | Non Allergen | HLA-DRB1*03:06,HLA-DRB1*03:07,HLA-DRB1*03:08,HLA-DRB1*03:05,HLA-DRB1*03:06,HLA-DRB1*03:07,HLA-DRB1*03:08,HLA-DRB1*03:05,HLA-DRB1*11:04,HLA-DRB1*11:06,HLA-DRB1*08:04,HLA-DRB1*08:13,HLA-DRB1*11:07,HLA-DRB1*11:28,HLA-DRB1*13:05,HLA-DRB1*13:11,HLA-DRB1*13:07,HLA-DRB1*13:21,HLA-DRB1*15:06 |
| 1225 | IAIVMVTIMLCCMTS | **0.744** | Non Allergen | HLA-DRB1*03:06,HLA-DRB1*03:07,HLA-DRB1*03:08,HLA-DRB1*03:05,HLA-DRB1*03:06,HLA-DRB1*03:07,HLA-DRB1*03:08,HLA-DRB1*03:05,HLA-DRB1*11:04,HLA-DRB1*11:06,HLA-DRB1*08:04,HLA-DRB1*08:13,HLA-DRB1*11:07,HLA-DRB1*11:28,HLA-DRB1*13:05,HLA-DRB1*13:11,HLA-DRB1*13:07,HLA-DRB1*13:21,HLA-DRB1*15:06 |
| 201 | FKIYSKHTPINLVRD | **0.729** | Non Allergen | HLA-DRB1*08:13,HLA-DRB1*11:20,HLA-DRB1*11:14,HLA-DRB1*13:23 |
| 1223 | GLIAIVMVTIMLCCM | **0.669** | Non Allergen | HLA-DRB1*03:06,HLA-DRB1*03:07,HLA-DRB1*03:08,HLA-DRB1*03:05,HLA-DRB1*03:06,HLA-DRB1*03:07,HLA-DRB1*03:08,HLA-DRB1*03:05,HLA-DRB1*11:04,HLA-DRB1*11:06,HLA-DRB1*08:04,HLA-DRB1*08:13,HLA-DRB1*11:07,HLA-DRB1*11:28,HLA-DRB1*13:05,HLA-DRB1*13:11,HLA-DRB1*13:07,HLA-DRB1*13:21,HLA-DRB1*15:06 |
| 820 | DLLFNKVTLADAGFI | **0.914** | Non Allergen | HLA-DRB1*08:17,HLA-DRB1*08:06 |
| 896 | IPFAMQMAYRFNGIG | **1.283** | Non Allergen | HLA-DRB1*13:01,HLA-DRB1*13:27,HLA-DRB1*13:29,DRB1_0101,DRB1_0102,DRB1_0103,DRB1_0104,DRB1_0105,DRB1_0106,DRB1_0107,DRB1_0108,DRB1_0109,DRB1_0110,DRB1_0112,DRB1_0113,DRB1_0114,DRB1_0115,DRB1_0118,DRB1_0119,DRB1_0120,DRB1_0122,DRB1_0123,DRB1_0125,DRB1_0126,DRB1_0127,DRB1_0128,DRB1_0129,DRB1_0130,DRB1_0131,DRB1_0132 |
| 628 | QLTPTWRVYSTGSNV | **0.928** | Non Allergen | HLA-DRB1*08:17,HLA-DRB1*08:06 |
| 900 | MQMAYRFNGIGVTQN | **1.303** | Non Allergen | HLA-DRB1*13:01,HLA-DRB1*13:27,HLA-DRB1*13:28 |
| 325 | SIVRFPNITNLCPFG | **0.79** | Non Allergen | HLA-DRB1*15:06,HLA-DRB1*15:02 |
| 822 | LFNKVTLADAGFIKQ | **0.557** | Non Allergen | HLA-DRB1*08:17,HLA-DRB1*08:06 |
| 104 | WIFGTTLDSKTQSLL | **0.524** | Non Allergen | HLA-DRB1*03:05,HLA-DRB1*03:09,HLA-DRB1*03:05,HLA-DRB1*03:09 |
| 628 | QLTPTWRVYSTGSNV | **0.928** | Non Allergen | HLA-DRB1*08:17,HLA-DRB1*08:06 |
| 2 | FVFLVLLPLVSSQCV | **0.719** | Non Allergen | HLA-DRB1*01:01,HLA-DRB1*01:01,HLA-DRB1*11:01,HLA-DRB1*11:02,HLA-DRB1*11:04,HLA-DRB1*11:06,HLA-DRB1*08:17,HLA-DRB1*11:28,HLA-DRB1*13:05,HLA-DRB1*11:21,HLA-DRB1*13:01,HLA-DRB1*11:14,HLA-DRB1*13:21,HLA-DRB1*15:02,HLA-DRB1*13:07,HLA-DRB1*13:22,HLA-DRB1*13:11,HLA-DRB1*13:27,HLA-DRB1*13:28,HLA-DRB1*13:23 |
| 325 | SIVRFPNITNLCPFG | **0.79** | Non Allergen | HLA-DRB1*15:06,HLA-DRB1*15:02 |
| 1060 | VVFLHVTYVPAQEKN | **1.172** | Non Allergen | HLA-DRB1*01:02,HLA-DRB1*04:02 |
| 885 | GWTFGAGAALQIPFA | **0.467** | Non Allergen | HLA-DRB1*01:01,HLA-DRB1*09:01,DRB1_0101,DRB1_0105,DRB1_0107,DRB1_0108,DRB1_0110,DRB1_0112,DRB1_0119,DRB1_0121,DRB1_0122,DRB1_0124,DRB1_0125,DRB1_0127,DRB1_0128,DRB1_0129,DRB1_0130,DRB1_0131,DRB1_0132 |

Table S10 Overlapping CTL epitopes in Membrane protein

| **Membrane Protein (CTL)** | | | | |
| --- | --- | --- | --- | --- |
| **Position** | **CTL** | **Antigenicity** | **Allergenicity** | **Supertypes/HLA Alleles** |
| 63 | **ACFVLAAVY** | **1.14** | Non Allergen | HLA-A*30:02,HLA-B*44:03,B62 |
| 65 | **FVLAAVYRI** | **0.514** | Non Allergen | HLA-A*02:01,HLA-A*02:06,HLA-A*23:01,HLA-A*68:02,HLA-A*32:01,HLA-B*51:01,HLA-B*53:01,A2 |
| 50 | **KLIFLWLLW** | **0.497** | Non Allergen | HLA-A*24:02,HLA-A*23:01,HLA-A*32:01,HLA-B*58:01,HLA-B*57:01,HLA-B*53:01,B58 |
| 62 | **LACFVLAAV** | **1.183** | Non Allergen | HLA-B*14:02,HLA-B*51:01 |
| 138 | **LVIGAVILR** | **1.103** | Non Allergen | HLA-A*11:01,HLA-A*68:01,HLA-A*31:01,A3 |
| 136 | **SELVIGAVI** | **0.641** | Non Allergen | HLA-B*40:01,HLA-B*40:02,HLA-B*44:02HLA-B*18:01,HLA-B*44:03,B44 |
| 94 | **SYFIASFRL** | **0.482** | Non Allergen | HLA-A*24:02,HLA-A*23:01,HLA-B*35:03,HLA-E*01:03,HLA-C*07:02,A24,B39 |
| 61 | **TLACFVLAA** | **1.193** | Non Allergen | HLA-A*02:01,A2 |
| 71 | **YRINWITGG** | **1.4** | Non Allergen | HLA-B*27:05,HLA-C*07:01,HLA-C*06:02,B27 |

Table S11 Overlapping HTL epitopes in Membrane protein

| **Membrane Protein (HTL)** | | | | |
| --- | --- | --- | --- | --- |
| **Position** | **HTL** | **Antigenicity** | **Allergenicity** | **Supertypes/HLA Alleles** |
| 61 | **TLACFVLAAVYRINW** | 1.3132 | Non Allergen | HLA-DRB1*01:02,HLA-DRB1*03:09,HLA-DRB1*03:05,HLA-DRB1*07:03,HLA-DRB1*07:01,HLA-DRB1*04:08,HLA-DRB1*11:20,HLA-DRB1*11:14,HLA-DRB1*11:28,HLA-DRB1*13:05,HLA-DRB1*13:23,HLA-DRB1*13:07 |
| 61 | **TLACFVLAAVYRINW** | 1.3132 | Non Allergen | HLA-DRB1*01:02,HLA-DRB1*03:09,HLA-DRB1*03:05,HLA-DRB1*07:03,HLA-DRB1*07:01,HLA-DRB1*04:08,HLA-DRB1*11:20,HLA-DRB1*11:14,HLA-DRB1*11:28,HLA-DRB1*13:05,HLA-DRB1*13:23,HLA-DRB1*13:07 |
| 47 | **YIIKLIFLWLLWPVT** | 0.4298 | Non Allergen | HLA-DRB1*08:01,HLA-DRB1*08:13,HLA-DRB1*08:17,HLA-DRB1*11:14,HLA-DRB1*13:23,HLA-DRB4*01:01 |
| 61 | **TLACFVLAAVYRINW** | 1.3132 | Non Allergen | HLA-DRB1*01:02,HLA-DRB1*03:09,HLA-DRB1*03:05,HLA-DRB1*07:03,HLA-DRB1*07:01,HLA-DRB1*04:08,HLA-DRB1*11:20,HLA-DRB1*11:14,HLA-DRB1*11:28,HLA-DRB1*13:05,HLA-DRB1*13:23,HLA-DRB1*13:07 |
| 136 | **SELVIGAVILRGHLR** | 0.6768 | Non Allergen | HLA-DRB1*03:09,HLA-DRB1*03:06,HLA-DRB1*03:07,HLA-DRB1*03:08,HLA-DRB1*11:07 |
| 136 | **SELVIGAVILRGHLR** | 0.6768 | Non Allergen | HLA-DRB1*03:09,HLA-DRB1*03:06,HLA-DRB1*03:07,HLA-DRB1*03:08,HLA-DRB1*11:07 |
| 88 | **VGLMWLSYFIASFRL** | 0.6658 | Non Allergen | DRB1_0906,DRB1_1501,DRB1_1503,DRB1_1506,DRB1_1507,DRB1_1509,DRB1_1510,DRB1_1512,DRB1_1513,DRB1_1516,DRB1_1518,DRB1_1520,DRB1_1522,DRB1_1523,DRB1_1524,DRB1_1529,DRB1_1532,DRB1_1533,DRB1_1536,DRB1_1540,DRB1_1541,DRB1_1542,DRB1_1543,DRB1_1545,DRB1_1546,DRB1_1548,HLA-DRB1*15:01 |
| 61 | **TLACFVLAAVYRINW** | 1.3132 | Non Allergen | HLA-DRB1*01:02,HLA-DRB1*03:09,HLA-DRB1*03:05,HLA-DRB1*07:03,HLA-DRB1*07:01,HLA-DRB1*04:08,HLA-DRB1*11:20,HLA-DRB1*11:14,HLA-DRB1*11:28,HLA-DRB1*13:05,HLA-DRB1*13:23,HLA-DRB1*13:07 |
| 71 | **YRINWITGGIAIAMA** | 1.1274 | Non Allergen | DRB1_0901,DRB1_0904,DRB1_0909,HLA-DRB1*03:09,HLA-DRB1*04:21 |

Table S12 Details of continuous B-cell epitopes present on the surface of target proteins

| **Proteins** | **Epitopes** | **Antigenicty Score** | **Antigenicty** | **Allergenicity** |
| --- | --- | --- | --- | --- |
| Surface | YAWNRKRISN | 0.5855 | Probable ANTIGEN | PROBABLE NON-ALLERGEN |
| Surface | FRKSNLKP | 1.1111 | Probable ANTIGEN | PROBABLE NON-ALLERGEN |
| Surface | GPKKSTNL | 0.7072 | Probable ANTIGEN | PROBABLE NON-ALLERGEN |
| Surface | LTESNKKF | 0.7708 | Probable ANTIGEN | PROBABLE NON-ALLERGEN |
| Surface | DEDDSEP | 0.6565 | Probable ANTIGEN | PROBABLE NON-ALLERGEN |
| Membrane | DIKDLPKEI | 0.5528 | Probable ANTIGEN | PROBABLE NON-ALLERGEN |
| Nucleocapsid | KEDLKFP | 0.9682 | Probable ANTIGEN | PROBABLE NON-ALLERGEN |
| Nucleocapsid | IKLDDKDPNFKDQ | 2.2233 | Probable ANTIGEN | PROBABLE NON-ALLERGEN |
| Nucleocapsid | PPTEPKKDKKKKADETQALPQRQKKQQTVT | 0.5132 | Probable ANTIGEN | PROBABLE NON-ALLERGEN |

Table S13 Details of Discontinuous B-cell epitopes present in target proteins

| **Protein** | **Residue** | **No. of Residue** | **Score** |
| --- | --- | --- | --- |
| Envelop | _:N64, _:L65, _:N66, _:S67, _:S68, _:R69, _:V70, _:P71, _:D72 | 9 | 0.84 |
| Nucleocapsid | _:M1, _:S2, _:D3, _:N4, _:G5, _:P6, _:Q7, _:N8, _:Q9, _:R10 | 10 | 0.98 |
|  | _:N11, _:A12, _:P13, _:R14, _:I15, _:T16, _:F17, _:G18, _:G19, _:P20, _:S21, _:D22, _:S23, _:T24, _:G25, _:S26, _:N27, _:Q28, _:N29, _:G30, _:E31 | 21 | 0.928 |
|  | _:M234, _:S235, _:G236, _:K237, _:G238, _:Q239, _:Q240, _:Q241, _:Q242, _:G243, _:Q244, _:T245, _:V246, _:T247, _:K248 | 15 | 0.864 |
|  | _:R93, _:R95, _:G96, _:G97, _:D98, _:G99, _:K100, _:M101 | 8 | 0.858 |
|  | _:V324, _:T325, _:P326, _:S327, _:G328, _:T329 | 6 | 0.849 |
|  | _:R32, _:S33, _:G34, _:A35, _:R36, _:S37, _:K38, _:Q39 | 8 | 0.842 |
|  | _:S190, _:R191, _:N192, _:S193, _:S194, _:R195, _:N196, _:S197, _:T198 | 9 | 0.828 |
| Surface | _:H1271, _:Y1272, _:T1273 | 3 | 0.998 |
|  | _:D1259, _:D1260, _:S1261, _:E1262, _:P1263, _:V1264, _:L1265, _:K1266, _:G1267, _:V1268, _:K1269, _:L1270 | 12 | 0.991 |
|  | _:L1234, _:C1235, _:C1236, _:M1237, _:T1238, _:S1239, _:C1240, _:C1241, _:S1242, _:C1243, _:L1244, _:K1245, _:G1246, _:C1247, _:C1248, _:S1249, _:C1250, _:G1251, _:S1252, _:C1253, _:C1254, _:K1255, _:F1256, _:D1257, _:E1258 | 25 | 0.967 |
|  | _:I1225, _:A1226, _:I1227, _:V1228, _:M1229, _:V1230, _:T1231, _:I1232, _:M1233 | 9 | 0.893 |
|  | _:M1, _:F2, _:V3, _:F4, _:L5, _:V6, _:Y707, _:S708, _:N709, _:N710, _:S711, _:N1074, _:F1075, _:T1076, _:T1077, _:A1078, _:P1079, _:A1080, _:I1081, _:C1082, _:H1083, _:D1084, _:G1085, _:K1086, _:A1087, _:H1088, _:G1093, _:F1095, _:V1096, _:S1097, _:N1098, _:G1099, _:T1100, _:H1101, _:W1102, _:F1103, _:V1104, _:T1105, _:Q1106, _:N1108, _:F1109, _:Y1110, _:E1111, _:P1112, _:Q1113, _:I1114, _:I1115, _:T1116, _:T1117, _:D1118, _:N1119, _:N1125, _:C1126, _:D1127, _:V1128, _:V1129, _:I1130, _:G1131, _:I1132, _:V1133, _:N1134, _:N1135, _:T1136, _:V1137, _:Y1138, _:D1139, _:P1140, _:L1141, _:Q1142, _:P1143, _:E1144, _:L1145, _:D1146, _:S1147, _:F1148, _:K1149, _:E1150, _:E1151, _:L1152, _:D1153, _:K1154, _:Y1155, _:F1156, _:K1157, _:N1158, _:H1159, _:T1160, _:S1161, _:P1162, _:D1163, _:V1164, _:D1165, _:L1166, _:G1167, _:D1168, _:I1169, _:S1170, _:G1171 | 98 | 0.872 |
|  | _:F329, _:P330, _:N331, _:I332, _:T333, _:N334, _:L335, _:C336, _:P337, _:F338, _:G339, _:E340, _:V341, _:N343, _:A344, _:T345, _:R346, _:F347, _:A348, _:S349, _:V350, _:Y351, _:A352, _:W353, _:N360, _:C361, _:V362, _:S373, _:F374, _:V401, _:I402, _:R403, _:K417, _:I418, _:Y421, _:N422, _:W436, _:N437, _:S438, _:N439, _:N440, _:L441, _:D442, _:S443, _:K444, _:V445, _:G446, _:G447, _:N448, _:Y449, _:N450, _:Y451, _:L452, _:Y453, _:R454, _:L455, _:F456, _:R457, _:K458, _:S459, _:N460, _:L461, _:E465, _:R466, _:D467, _:I468, _:S469, _:T470, _:E471, _:I472, _:Y473, _:Q474, _:A475, _:G476, _:S477, _:T478, _:P479, _:C480, _:N481, _:G482, _:V483, _:E484, _:G485, _:F486, _:N487, _:C488, _:Y489, _:F490, _:P491, _:L492, _:Q493, _:S494, _:Y495, _:G496, _:F497, _:Q498, _:P499, _:T500, _:N501, _:G502, _:V503, _:G504, _:Y505, _:Q506, _:P507, _:Y508, _:R509 | 107 | 0.871 |
|  | _:G838, _:D839, _:C840, _:L841, _:G842, _:D843, _:I844, _:A845 | 8 | 0.868 |
|  | _:F833, _:I834, _:K835, _:Q836, _:Y837 | 5 | 0.86 |
|  | _:A684, _:R685, _:S686, _:V687 | 4 | 0.801 |
| Membrane | _:H210, _:S211, _:S212, _:S213, _:S214, _:D215, _:N216, _:I217, _:A218, _:L219, _:L220, _:V221, _:Q222 | 13 | 0.939 |
|  | _:M1, _:A2, _:D3, _:S4, _:N5, _:G6, _:T7, _:I8 | 8 | 0.912 |
|  | _:V70, _:Y71, _:R72, _:I73, _:N74, _:W75, _:I76 | 7 | 0.842 |
|  | _:A152, _:G153, _:H154, _:H155, _:L156 | 5 | 0.81 |

Table S14 List of highest antigenic IFN-gamma epitopes predicted by IFNepitope server

| **IFN ID** | **Protein** | **Position** | **Epitope** | **Method** | **Result** | **Score** | **Antigenicity Score** | **Antigenicity** | **Allergenicity** |
| --- | --- | --- | --- | --- | --- | --- | --- | --- | --- |
| IFN1 | Envelop | 37 | RLCAYCCNIVNVSLV | MERCI | Positive | 1 | 1.2823 | Probable Antigen | Probable Non Allergen |
| IFN2 | Membrane | 60 | TLACFVLAAVYRINW | SVM | Positive | 0.04691397 | 1.3132 | Probable Antigen | Probable Non Allergen |
| IFN3 | Nucleocapsid | 99 | KMKDLSPRWYFYYLG | SVM | Positive | 0.39401059 | 1.4297 | Probable Antigen | Probable Non Allergen |
| IFN4 | Surface | 25 | TFKCYGVSPTKLNDL | MERCI | Positive | 1 | 1.4626 | Probable Antigen | Probable Non Allergen |

Table S15 Details of overlapping CTL+HTL epitopes with their population coverage efficiency

| **S.No** | **Protein** | **Overlapping CTL+HTL** | **Population coverage** |
| --- | --- | --- | --- |
| 1 | Envelop | LLFLAFVVFLLVTLA | 87.31% |
| 2 | Envelop | LAFVVFLLVTLAILT | 91.14% |
| 3 | Envelop | NIVNVSLVKPSFYVY | 84.46% |
| 4 | Envelop | SVLLFLAFVVFLLVT | 54.74% |
| 5 | Envelop | VKPSFYVYSRVKNLN | 51.57% |
| 6 | Membrane | TLACFVLAAVYRINW | 69.44% |
| 7 | Membrane | VGLMWLSYFIASFRL | 87.07% |
| 8 | Nucleocapsid | GDAALALLLLDRLNQ | 77.89% |
| 9 | Nucleocapsid | AQFAPSASAFFGMSR | 54.44% |
| 10 | Surface | IPFAMQMAYRFNGIG | 65.21% |
| 11 | Surface | FVFLVLLPLVSSQCV | 71.52% |
| 12 | Surface | LFNKVTLADAGFIKQ | 81.83% |
| 13 | Surface | WIFGTTLDSKTQSLL | 87.25% |
| 14 | Surface | VFLHVTYVPAQEKNF | 58.14% |
| 15 | Surface | DPFLGVYYHKNNKSW | 75.98% |

Table S16 List of discontinuous epitopes predicted over final vaccine construct

| **No.** | **Residues** | **Number of residues** | **Score** |
| --- | --- | --- | --- |
| 1 | _:D21, _:L22, _:A23, _:K24, _:I25, _:L26, _:K27, _:E28, _:K29, _:Y30, _:G31, _:L32, _:D33, _:P34, _:S35, _:A36, _:N37, _:L38, _:A39, _:I40, _:P41 | 21 | 0.93 |
| 2 | _:M1, _:S2, _:D3, _:I4, _:N5, _:K6, _:L7, _:A8, _:E9, _:T10, _:L11, _:V12, _:N13, _:L14, _:K15, _:E18 | 16 | 0.915 |
| 3 | _:G291, _:P292, _:G293, _:P294, _:G295, _:A296, _:Q297, _:F298, _:A299, _:P300, _:S301, _:A302, _:S303, _:F306, _:G307 | 15 | 0.869 |
| 4 | _:T256, _:C259, _:F260 | 3 | 0.85 |
| 5 | _:A163, _:A164, _:G166, _:D167, _:A168, _:A171, _:L172 | 7 | 0.843 |
| 6 | _:L344, _:V345, _:S346, _:S347, _:Q348, _:C349, _:V350, _:G351, _:P352, _:G353, _:P354, _:G355, _:R356 | 13 | 0.819 |

Table S17 Cluster scores of docked vaccine TLR3 complex

| **Cluster** | **Members** | **Representative** | **Weighted Score** |
| --- | --- | --- | --- |
| **1** | 60 | Center | -1025.7 |
|  |  | Lowest Energy | -1049.5 |
| **2** | 46 | Center | -1010.5 |
|  |  | Lowest Energy | -1199.1 |
| **3** | 34 | Center | -978.4 |
|  |  | Lowest Energy | -1086.7 |
| **4** | 32 | Center | -951.4 |
|  |  | Lowest Energy | -1183.7 |
| **5** | 30 | Center | -948.4 |
|  |  | Lowest Energy | -1149.0 |
| **6** | 29 | Center | -932.9 |
|  |  | Lowest Energy | -1093.4 |
| **7** | 28 | Center | -1036.5 |
|  |  | Lowest Energy | -1079.9 |
| **8** | 23 | Center | -933.7 |
|  |  | Lowest Energy | -1101.4 |
| **9** | 22 | Center | -938.2 |
|  |  | Lowest Energy | -1161.2 |
| **10** | 22 | Center | -1089.1 |
|  |  | Lowest Energy | -1089.1 |
| **11** | 21 | Center | -1078.1 |
|  |  | Lowest Energy | -1078.1 |
| **12** | 20 | Center | -980.6 |
|  |  | Lowest Energy | -1097.7 |
| **13** | 20 | Center | -1160.8 |
|  |  | Lowest Energy | -1231.7 |
| **14** | 19 | Center | -977.5 |
|  |  | Lowest Energy | -1036.0 |
| **15** | 16 | Center | -1075.9 |
|  |  | Lowest Energy | -1182.9 |
| **16** | 16 | Center | -1083.1 |
|  |  | Lowest Energy | -1149.6 |
| **17** | 16 | Center | -1156.0 |
|  |  | Lowest Energy | -1156.0 |
| **18** | 16 | Center | -1009.4 |
|  |  | Lowest Energy | -1050.4 |
| **19** | 15 | Center | -964.6 |
|  |  | Lowest Energy | -1066.2 |
| **20** | 15 | Center | -1044.5 |
|  |  | Lowest Energy | -1044.5 |
| **21** | 13 | Center | -957.5 |
|  |  | Lowest Energy | -1163.1 |
| **22** | 13 | Center | -1080.4 |
|  |  | Lowest Energy | -1080.4 |
| **23** | 12 | Center | -951.6 |
|  |  | Lowest Energy | -1166.7 |
| **24** | 12 | Center | -946.8 |
|  |  | Lowest Energy | -991.1 |
| **25** | 11 | Center | -933.3 |
|  |  | Lowest Energy | -992.3 |
| **26** | 11 | Center | -1032.7 |
|  |  | Lowest Energy | -1176.4 |
| **27** | 10 | Center | -985.9 |
|  |  | Lowest Energy | -1002.7 |
| **28** | 10 | Center | -940.0 |
|  |  | Lowest Energy | -1037.6 |
| **29** | 9 | Center | -969.3 |
|  |  | Lowest Energy | -1043.0 |
| **30** | 8 | Center | -970.5 |
|  |  | Lowest Energy | -989.5 |

Table S18 Cluster scores of docked vaccine TLR4 complex

| **Cluster** | **Members** | **Representative** | **Weighted Score** |
| --- | --- | --- | --- |
| **1** | 79 | Center | -982.5 |
|  |  | Lowest Energy | -1229.9 |
| **2** | 47 | Center | -1164.2 |
|  |  | Lowest Energy | -1164.2 |
| **3** | 45 | Center | -1028.2 |
|  |  | Lowest Energy | -1112.5 |
| **4** | 45 | Center | -1016.5 |
|  |  | Lowest Energy | -1127.0 |
| **5** | 44 | Center | -948.4 |
|  |  | Lowest Energy | -1104.8 |
| **6** | 44 | Center | -933.7 |
|  |  | Lowest Energy | -1132.8 |
| **7** | 42 | Center | -932.4 |
|  |  | Lowest Energy | -1172.4 |
| **8** | 31 | Center | -914.5 |
|  |  | Lowest Energy | -1084.2 |
| **9** | 28 | Center | -1152.3 |
|  |  | Lowest Energy | -1328.3 |
| **10** | 27 | Center | -930.1 |
|  |  | Lowest Energy | -1182.1 |
| **11** | 25 | Center | -933.4 |
|  |  | Lowest Energy | -1071.1 |
| **12** | 25 | Center | -956.7 |
|  |  | Lowest Energy | -1232.9 |
| **13** | 24 | Center | -1040.2 |
|  |  | Lowest Energy | -1040.2 |
| **14** | 24 | Center | -931.8 |
|  |  | Lowest Energy | -1024.3 |
| **15** | 23 | Center | -904.7 |
|  |  | Lowest Energy | -1130.5 |
| **16** | 23 | Center | -963.2 |
|  |  | Lowest Energy | -1034.7 |
| **17** | 20 | Center | -963.9 |
|  |  | Lowest Energy | -1140.5 |
| **18** | 20 | Center | -987.0 |
|  |  | Lowest Energy | -1204.1 |
| **19** | 19 | Center | -1078.2 |
|  |  | Lowest Energy | -1078.2 |
| **20** | 17 | Center | -950.5 |
|  |  | Lowest Energy | -1031.0 |
| **21** | 14 | Center | -904.0 |
|  |  | Lowest Energy | -1071.0 |
| **22** | 14 | Center | -1017.0 |
|  |  | Lowest Energy | -1017.0 |
| **23** | 13 | Center | -1052.1 |
|  |  | Lowest Energy | -1052.1 |
| **24** | 13 | Center | -961.7 |
|  |  | Lowest Energy | -1202.8 |
| **25** | 13 | Center | -956.3 |
|  |  | Lowest Energy | -1129.9 |
| **26** | 12 | Center | -1027.4 |
|  |  | Lowest Energy | -1028.6 |
| **27** | 11 | Center | -973.7 |
|  |  | Lowest Energy | -1165.3 |
| **28** | 10 | Center | -933.4 |
|  |  | Lowest Energy | -1051.7 |
| **29** | 10 | Center | -995.1 |
|  |  | Lowest Energy | -1055.0 |
| **30** | 9 | Center | -975.2 |
|  |  | Lowest Energy | -1056.4 |

**Table S19. Comparison of experimentally determined T-cell epitopes of SARS-CoV and SARS-CoV2**

| **T-cell Epitopes** | | | | | | | | |
| --- | --- | --- | --- | --- | --- | --- | --- | --- |
| **Experimentally determined** | | | | | **Predicted epitopes** | | | |
| **Nucleocapsid** | **Antigenicity Score** | | **Probable Antigenicity** | **Probable Allergenicity** | **Nucleocapsid** | **Antigenicity Score** | **Probable Antigenicity** | **Probable Allergenicity** |
| AFFGMSRIGMEVTPSGTW | 0.7982 | | Antigen | Non Allergen | ASAFFGMSRIGMEVT | 0.862 | Antigen | Non Allergen |
| MEVTPSGTWL | 0.6342 | | Antigen | Non Allergen | ASAFFGMSRIGMEVT | 0.862 | Antigen | Non Allergen |
| GMSRIGMEV | 0.6287 | | Antigen | Non Allergen | ASAFFGMSRIGMEVT | 0.862 | Antigen | Non Allergen |
| LALLLLDRL | 0.5933 | | Antigen | Non Allergen | LALLLLDRLNQLESK | 0.7357 | Antigen | Non Allergen |
| AQFAPSASAFFGMSR | 0.5266 | | Antigen | Non Allergen | AQFAPSASAFFGMSR | 0.5266 | Antigen | Non Allergen |
| AQFAPSASAFFGMSRIGM | 0.7461 | | Antigen | Non Allergen | AQFAPSASAFFGMSR | 0.5266 | Antigen | Non Allergen |
| **Surface** |  | |  |  | **Surface** |  |  |  |
| VVFLHVTYV | 1.5122 | | Antigen | Non Allergen | GVVFLHVTYVPAQEK | 1.1043 | Antigen | Non Allergen |
| VVFLHVTYV | 1.5122 | | Antigen | Non Allergen | VVFLHVTYVPAQEKN | 1.172 | Antigen | Non Allergen |
| **Linear B- cell epitopes** | | | | | | | | |
| **Nucleocapsid** | **Antigenicity Score** | **Probable Antigenicity** | | **Probable Allergenicity** | **Nucleocapsid** | **Antigenicity Score** | **Probable Antigenicity** | **Probable Allergenicity** |
| KTFPPTEPKKDKKKK | 0.3767 | Non Antigen | | Non Allergen | PPTEPKKDKKKKADETQALPQRQKKQQTVT | 0.5132 | Antigen | Non Allergen |
| LPQRQKKQ | 1.3573 | Antigen | | Non Allergen | PPTEPKKDKKKKADETQALPQRQKKQQTVT | 0.5132 | Antigen | Non Allergen |
| **Discontinuous B- cell epitopes** | | | | | |  |  |  |
| **Experimentally determined** | | | | | **Predicted epitopes** | | | |
| **Surface** | **Antigenicity Score** | **Probable Antigenicity** | | **Probable Allergenicity** | **Surface** | **Antigenicity Score** | **Probable Antigenicity** | **Probable Allergenicity** |
| G446 | Na | Na | | Na | G446 | Na | Na | Na |
| G482 | Na | Na | | Na | G482 | Na | Na | Na |
| N437 | Na | Na | | Na | N437 | Na | Na | Na |
| G482 | Na | Na | | Na | G482 | Na | Na | Na |
